# vB_BcM_Sam46 and vB_BcM_Sam112, members of a new bacteriophage genus with unusual small terminase structure

**DOI:** 10.1038/s41598-021-91289-x

**Published:** 2021-06-09

**Authors:** Olesya A. Kazantseva, Emma G. Piligrimova, Andrey M. Shadrin

**Affiliations:** Laboratory of Bacteriophage Biology, G.K. Skryabin Institute of Biochemistry and Physiology of Microorganisms, Pushchino Scientific Center for Biological Research of the Russian Academy of Sciences, Federal Research Center, 142290 Pushchino, Russia

**Keywords:** Phage biology, Virology

## Abstract

One of the serious public health concerns is food contaminated with pathogens and their vital activity products such as toxins. *Bacillus cereus* group of bacteria includes well-known pathogenic species such as *B. anthracis, B. cereus sensu stricto (ss)*, *B. cytotoxicus* and *B. thuringiensis*. In this report, we describe the *Bacillus* phages vB_BcM_Sam46 and vB_BcM_Sam112 infecting species of this group. Electron microscopic analyses indicated that phages Sam46 and Sam112 have the myovirus morphotype. The genomes of Sam46 and Sam112 comprise double-stranded DNA of 45,419 bp and 45,037 bp in length, respectively, and have the same GC-content. The genome identity of Sam46 and Sam112 is 96.0%, indicating that they belong to the same phage species. According to the phylogenetic analysis, these phages form a distinct clade and may be members of a new phage genus, for which we propose the name ‘*Samaravirus*’. In addition, an interesting feature of the Sam46 and Sam112 phages is the unusual structure of their small terminase subunit containing N-terminal FtsK_gamma domain.

## Introduction

Bacteriophages, also known as phages, are viruses that infect bacteria. It is well established that phages are the most abundant biological organisms, with a total amount of about $10^{30}$–$10^{32}$ phage particles on the planet^1^. Bacteriophages are ubiquitous in all habitats of their prokaryotic hosts, including extreme ones^2–5^. Phages are known to play a significant role in biospheric processes. They are involved in global biogeochemical cycling (such as the carbon and nitrogen cycles) and ecology by regulating the abundance and composition of microbial communities^6–8^. Bacteriophages are the important part of genetic exchange in the environment and actively influence microbial evolution by means of generalized, specialized and lateral transduction^9,10^. However, phages are of considerable interest not only for fundamental biology but also for medicine and biotechnology. On the one hand, bacteriophages are microbial predators and can be used as a preventive measure or treatment for bacterial infections, which are a serious hazard to both human health and activities such as agriculture, animal husbandry and food industry. This fact offers incredible prospects for R&D projects and drug design based on phages and/or their lytic proteins (endolysins and virion-associated peptidoglycan hydrolases)^11^. To date, phages have been applied as sources of diagnostic and genetic tools and as novel therapeutic agents^12–14^. On the other hand, it should be remembered that phage transduction is one of the mechanisms of horizontal gene transfer (HGT) between bacterial cells^9,10^. Therefore, bacteriophages can be potential sources of virulence factors and antibiotic resistance of bacteria^15–17^. Thereby, studying of phage diversity and role in the evolution and life of bacterial hosts is of great significance for improvement and extension of the scope of phage application, in particular, in medicine and biotechnology.

One of the serious public health concerns is food contaminated with pathogens and their vital activity products such as toxins. The *Bacillus cereus* group, equally well-known as *B. cereus sensu lato (sl)*, is a group of Gram-positive spore-forming bacteria, which comprises genetically closely related species with rather broad-spectrum pathogenicity. The most remarkable members of this group are *B. anthracis*, *B. cereus sensu stricto (ss)*, *B. cytotoxicu*s and *B. thuringiensis*. *B. anthracis* is an obligate pathogen and causes anthrax, an acute infectious disease that mainly infects livestock and wild ungulates, but anthrax outbreaks occur in humans annually and require constant monitoring and risk assessment^18^. *B. cereus* is an opportunistic pathogen and is associated with two types of foodborne poisonings, in particular, the diarrheal syndrome caused by enterotoxins and the emetic syndrome caused by the toxin cereulide^19^. *B. cytotoxicus* is the less studied species than those mentioned above inasmuch as it has been fairly recently described for the first time^20^. *B. cytotoxicus* is responsible for severe diarrheal disease due to production of its *cytK*2 variant of cytotoxin K, which is able to cause necrotic enteritis^21^. Contamination of foodstuffs with spores and/or vegetative cells of both *B. cereus* and *B. cytotoxicus* poses a serious threat to public health and can cause extensive harm to food industry and agriculture. *B. thuringiensis* is referred to as an entomopathogenic species able to produce crystal insecticidal toxins ($\delta$-endotoxins)^22^. This is why biopesticidal strains of *B. thuringiensis* have become widespread as microbiological biopesticides being the most environmentally friendly and safe for human health among insecticidal products available^23,24^. Nevertheless, the actual contribution of *B. thuringiensis* to the cases of poisoning is still highly contentious^25,26^.

Taking into account the major challenge of the emergence and spread of multi-drug resistant bacteria including pathogens of the *B. cereus* group^27,28^, it is apparent that phage therapy has the potential to become either an alternative or a supplement to the classical antibiotic treatments. However, the creation of effective phage-based preparations requires developing and maintaining the collections of carefully selected and well-studied bacteriophages acting on target pathogen species and strains.

In this study, we describe the newly isolated and characterized *Bacillus* phages vB_BcM_Sam46 and vB_BcM_Sam112 (abbreviated as Sam46 and Sam112, respectively), which effectively infect members of the *B. cereus* group. Phages Sam46 and Sam112 have morphological characteristics typical of myoviruses. The genomes of Sam46 and Sam112 comprise double-stranded DNA, 45,419 bp and 45,037 bp in length, respectively, and the same GC-content. The genome identity of Sam46 and Sam112 is 96.0% indicating that they belong to the same phage species. According to the phylogenetic analysis, these phages form a distinct clade and may be members of a new phage genus. We propose to create a new bacteriophage genus called ‘*Samaravirus*’ to formally classify these phages.

## Materials and methods

### Bacterial strains and growth conditions

The bacterial strains used in this study were obtained from the All-Russian Collection of Microorganisms (VKM). Lysogeny broth (LB) and LB agar (1.5% w/v and 0.75% w/v) with 10 mM CaCl$_{2}$ and 10 mM MgCl$_{2}$ were used for bacterial and phage cultivation. All cultures were grown at 37 $^\circ$C.

### Phage isolation and propagation

Phages Sam46 and Sam112 were isolated from soil samples collected in Samara, Russian Federation, and propagated on the sensitive strain *B. cereus* VKM B-370. The sensitive strain *B. cereus* VKM B-370 was grown in 10 ml of LB broth with 10 mM CaCl$_{2}$ and 10 mM MgCl$_{2}$ to the optical density of 0.6 at 590 nm. One gram of the soil sample was added to the cell culture and the mixture was incubated for 2 h at 37 $^\circ$C until optical density decreased. The obtained suspension was centrifuged for 10 min at 12,000*g*. After that, the lysate was mixed with 100 $\upmu$l of *B. cereus* VKM B-370 culture (OD590 of 0.35) and 1.5% agar to the final agar concentration of 0.5%. After gentle short-term vortexing, the mixture was poured into Petri dishes with previously prepared LB agar (1.5%) and incubated overnight at 37 $^\circ$C. Further, the separate plaques were transferred into 2 ml of SM+ buffer [125 mM Tris-HCl, pH 8.0; 100 mM NaCl; 2.5 mM MgSO$_{4}$; 0.01% gelatin; 10 mM CaCl$_{2}$] and incubated overnight at 4 $^\circ$C with shaking for phage extraction. The extracts of single plaques were centrifuged for 10 min at 12,000*g*, and 50 $\upmu$l of the supernatants were transferred into a 48-well plate containing 500 $\upmu$l of LB broth (with additional 10 mM CaCl$_{2}$, 10 mM MgCl$_{2}$) with 5 $\upmu$l of *B. cereus* VKM B-370 culture (OD590 0.6) and incubated for 16 h at 37 $^\circ$C until optical density decreased. The cultures with the lowest optical densities were transferred into 1.5 ml Eppendorf tubes with 50 $\upmu$l of chloroform. The cell debris was removed by centrifugation for 10 min at 12,000*g*. The obtained lysates were titrated by serial dilutions. In order to exclude the presence of other phages with morphologically identical plaques, the extraction-titration cycles were repeated five times. Phage propagation and PEG 8,000 (polyethylene glycol) precipitation were performed as described previously^29^. The obtained high-titer phage sample was filtered through a 0.22 $\upmu$m sterile filter and stored at 4 $^\circ$C. Three ml of the high-titer preparation was subsequently used to prepare the final purified phage suspension by CsCl density gradient centrifugation (with preformed gradient of CsCl: 1.3 g/ml, 1.4 g/ml, 1.5 g/ml, 1.6 g/ml and 1.7 g/ml). The purified suspension was ultimately used for transmission electron microscopy.

### Host range determination

A host range test was performed using 32 strains of the *B. cereus* group as described previously^29^ with differences in incubation conditions (for 24 h at 37 $^\circ$C).

### Transmission electron microscopy

Phage suspension applied onto 400 mesh carbon-formvar coated copper grids was negatively stained with 1% uranyl acetate and subsequently analyzed using a JEM-100C (JEOL, Japan) transmission electron microscope at 80 kV accelerating voltage. Images were taken on Kodak film SO-163 (Kodak, Cat. # 74144, Hatfield, PA, USA). Phage particle dimensions were measured using ImageJ version 1.53e in relation to the scale bar generated from the microscope.

### Genome sequencing, assembly and sequence analysis

Phage DNA was extracted using the standard phenol-chloroform extraction protocol described by Sambrook et al.^30^. Purified phage DNA was sequenced using Illumina with TruSeq library preparation technology. The de novo genome assembly was accomplished by SPAdes v.3.11.1 software^31^ . Open reading frames (ORFs) were identified by RASTtk v.2.0^32^. The putative functions were predicted using BLAST (NCBI)^33^ and HHpred^34^. The circular genome visualizations were created by BRIG software v.0.95^35^.

### Sequence alignment analysis and Neighbor-Net network analysis

The accessions of the analyzed FtsK gamma domain were as follows: Sam46: QIQ61202.1, 11–70 aa; Sam112: QGF21705.1, 11–70 aa; *B. cereus*: SME41550.1, 727–786 aa; *B. subtilis*: WP_003231869, 721–780 aa; *E. coli*: NP_415410.1, 1266–1325 aa; *L. lactis*: WP_003129457.1, 693–752 aa; *P. aeruginosa*: ARG86759.1, 747–806 aa. The dataset sequence was used for alignment by MAFFT version 7 with default options^36^. Alignment data were manually trimmed and the aligned residues of the FtsK_gamma domain (60 aa in length) were used for the Neighbor-Net network analysis. The Neighbor-Net network tree was drawn by SplitTree4 v.4.16.1^37^ with 1000 bootstrap replicates.

### Comparative genomics

To assess the phylogenetic relationship of Sam46 and Sam112 to the known phages, ViPTree server version 1.9^38^ was used to generate a proteomic tree based on the genome-wide sequence similarities computed by tBLASTx. The linear comparison diagram showing the similarity between the most closely related phage genomes was visualized with the ViPTree server version 1.9^38^. The number of shared proteins was computed using the GET_HOMOLOGUES software v3.3.3^39^ with the COGtriangles algorithm^40^ (-t 0 –C 75 -e). The compare_clusters.sh script from the GET_HOMOLOGUES package was used to produce a pangenome matrix showing the presence/absence of representatives of individual protein clusters in each genome. The matrix was then used to build a Maximum-likelihood tree with the GET_PHYLOMARKERS software package version 2.2.8.1^41^. The resulting tree was rooted at the midpoint and visualized with FigTree v1.4.4^42^.

### Headful DNA analysis

The bacteriophage genome termini were identified based on the read occurrence frequency following the High Occurrence Read Termini theory^43,44^. Mapping the reads to the genome was carried out with the Bowtie2 software tool v.2.3.4.3^45,46^. The in silico results were experimentally confirmed using a standard restriction analysis^47^. The *pac*-site was determined by sequencing terminal DNA fragments obtained by the method of rapid amplification of genomic ends (RAGE)^48^, which is based on the principles of the 5$'$-Rapid amplification of cDNA ends (5$'$-RACE). For this purpose, the specific fragments suspected to contain the phage genome termini were extracted from electrophoresis gel after restriction analysis and used for a typical DNA tailing reaction by terminal transferase (New England Biolab, Cat. # M0315L). Further, two PCRs were carried out consecutively using the TaqSE DNA polymerase (SibEnzyme, Cat. # E314) and the pairs of oligonucleotides Fw-d(T)16V 5$'$-GACCACGCGTATCGATGTCGACTTTTTTTTTTTTTTTTV-3$'$ plus Rv-1 5$'$-AACTAATTCGTCGCTGCTCATA-3$'$ and Fw-d(T)16V 5$'$-GACCACGCGTATCGATGTCGACTTTTTTTTTTTTTTTTV-3$'$ plus Rv-2 5$'$-CTCAATTGCTGCCGCTGTG-3$'$, respectively. The final PCR products were extracted from electrophoresis gel and used for Sanger sequencing with primer Rv-2.

### Identification of genetic differences between phages with turbid and clear plaque morphotypes

In order to identify genetic differences between the Sam46 types forming turbid and clear plaques (Sam46-T and Sam46-C, respectively), separate plaques with different morphology were used for T-type and C-type phage extraction followed by obtaining high-titer phage samples, phage DNA extraction and whole genome sequencing (WGS) as mentioned above.

To obtain mutant phages Sam46-C and Sam112-C from Sam46-T and Sam112-T, respectively, T-type phages were propagated in 30 ml of LB broth (10 mM CaCl$_{2}$, 10 mM MgCl$_{2}$) inoculated with 300 $\mu$l of *B. cereus* VKM B-370 culture (OD590 0.6). Cultivation was continued at 37 $^\circ$C until optical density decreased. The cell debris was removed by centrifugation at 12,000*g* for 10 min at 4 $^\circ$C. Phage particles were precipitated from the obtained lysates with PEG 8,000 as described previously^29^. The propagation–precipitation cycles were repeated three times. Then, 10 $\upmu$l of the final concentrated T-type phage suspensions diluted to $10^{4}$ plaque-forming units (PFU)/ml were mixed with 100 $\upmu$l of *B. cereus* VKM B-370 culture (OD590 0.6) and 5 ml of LB top agar (0.75%) and overlaid on LB agar plates (1.5% w/v agar) in Petri dishes. Incubation was performed at 37 $^\circ$C overnight for the formation of phage plaques and detection of turbid-to-clear plague mutations. Thus, 40 plates were analyzed for each T-type phage (Sam46-T and Sam112-T). For each phage, three clear plaques were selected for further manipulations. The selected mutated plaques were separately transferred into 50 $\upmu$l of Milli-Q water and incubated for 15 min at 95 $^\circ$C. Two microliters of each mixture were used as PCR templates. PCRs were carried out consecutively using the TaqSE DNA polymerase (SibEnzyme, Cat. # E314) and the pair of oligonucleotides: Sam46-112_CDS_24_Fw 5$'$-TCTATTTTCAAAGCAAGCGG-3$'$ and Sam46-112_CDS_26_Rv 5$'$-GCTAATTTCTTAACCGGTTC-3$'$. PCR products were extracted from electrophoresis gel and used for Sanger sequencing with the primer Sam46-112_CDS_24_Fw 5$'$-TCTATTTTCAAAGCAAGCGG-3$'$.

The phage mutants with turbid plaques from Sam46-C and Sam112-C were obtained similarly.

### Prediction of protein secondary structures

Secondary structure prediction of the gp25 gene products of Sam46 and Sam112 phages was performed with JPRED4 incorporating the Jnet algorithm v2.3.1^49^. The oligomeric state probabilities of a coiled-coil sequence were predicted by LOGICOIL algorithm^50^.

### Thermal and pH stability of the Sam46-T and Sam46-C phages

In order to assess the stability of Sam46-T and Sam46-C phages at various temperatures, aliquots of phage suspensions at a titer of $5\times 10^8$ plaque-forming units (PFU)/ml were incubated at 4, 30, 40, 50, 60, 70, 80, 90 $^\circ$C for 1 h. In addition, phage stability at pH values ranging from 2.2 to 10 was determined using three different buffers: SM+ buffer (pH values 6–10), sodium acetate buffer (pH 4 and 5), and Glycine-HCl buffer (pH 2.2 and 3). The Sam46-T and Sam46-C phages were added to each pH solution to the final concentration of $5\times 10^8$ and incubated for 1 h at 37 $^\circ$C. The surviving phages from both thermal and pH stability experiments were enumerated by double-layer plate titration. The experiment was performed with three replicates. The results were visualized in SigmaPlot v.12.5 as the mean of three observations ± standard deviation.

### Killing assay

To assess the killing activity of the Sam46-C and Sam46-T phages, the *B. cereus* VKM B-370 strain was separately infected with the Sam46-C and Sam46-T phages at different multiplicity of infection (MOI) values. Briefly, 50 $\upmu$l of phage suspensions ($2\times 10^9$, $2\times 10^8$ and $2\times 10^7$ PFU/ml) were mixed separately in a 48-well microplate with 450 $\upmu$l of mid-log *B. cereus* VKM B-370 culture at a concentration of $2\times 10^7$ colony-forming units (CFU)/ml to provide MOI values of 10, 1, and 0.1, respectively. The microplate was incubated with shaking for 7 h at 30 $^\circ$C in a FilterMax F5 microplate reader (Molecular Devices), with OD595 being measured every 10 min. Non-infected *B. cereus* VKM B-370 culture was used as a control sample. The experiment was performed with three replicates. The results were reported as the mean of three observations ± standard deviation. The obtained growth curves were visualized in SigmaPlot v.12.5.

### Adsorption assay

To determine the time required for the Sam46-T and Sam46-C phages to attach to *B. cereus* VKM B-370 cells, an adsorption assay was performed according to the protocol developed by Kropinski^51^. Briefly, 0.95 ml of LB broth with three drops of chloroform were added to Eppendorf tubes and placed on ice to chill for 10 min. Mid-log phase *B. cereus* VKM B-370 culture (OD590 0.4) grown in LB broth with the addition of 10 mM CaCl$_{2}$ and 10 mM MgCl$_{2}$ was diluted to OD590 of 0.2. Nine milliliters of the culture were transferred to a 100-ml laboratory flask and the flask was placed into a shaking water bath at 37 $^\circ$C, 60 rpm. Nine milliliters of LB broth with 10 mM CaCl$_{2}$ and 10 mM MgCl$_{2}$ were used as a control sample. Immediately, 1 ml of the Sam46-T or Sam46-C phage suspension at a concentration of $2\times 10^{5}$ PFU/ml (preheated for 5 min at 37 $^\circ$C) was added to both tested and control flasks. Every 5 min, 50-μl aliquots were collected from both tested and control flasks, transferred into the prepared Eppendorf tubes containing LB and chloroform, and mixed vigorously in a vortex mixer. The mixtures were assayed for unadsorbed phages by double-layer plate titration, and the resulting phage titers were compared to those obtained for the control samples (without host cells). This experiment was performed three times for each phage (Sam46-T and Sam46-C). The results were presented as percentages of the initial phage number and visualized in SigmaPlot v.12.5 with error bars representing standard deviation for three replicates. The adsorption rate was calculated using the equation described by Kropinski^51^.

### One-step growth curve

In order to determine the latent period and the average burst size of the Sam46-T and Sam46-C phages, the one-step growth experiment was performed as described by Hyman and Abedon^52^. Briefly, 1ml of phage suspension (preheated for 5 min at 37 $^\circ$C) at a concentration of $2\times 10^{7}$ was mixed with 9 ml of the *B. cereus* VKM B-370 culture grown to the mid-log phase (OD590 0.4) and diluted to OD590 of 0.2 to provide MOI of 0.1. After the incubation at 37 $^\circ$C for 10 min for phage adsorption, a 1-ml aliquot was collected and centrifuged at 3,500*g* for 10 min at 4 $^\circ$C to precipitate the cells. The supernatant was removed and the pellet was resuspended in 1 ml of fresh LB broth, centrifuged and resuspended in 1 ml of LB again. 0.1 ml of the mixture (bacteria with adsorbed phages) was transferred to 9.9 ml of LB growth medium in a 100-ml flask, mixed thoroughly and placed in a shaking water bath (60 rpm) at 37 $^\circ$C. Aliquots of 150 $\upmu$l of the mixture were collected at 5-min intervals for 1 h. The phages were enumerated by double-layer plate titration at each time point, including time point 0. The experiment was performed in triplicate for each phage (Sam46-T and Sam46-C). The PFU/ml values were calculated and plotted against time. The latent period and phage burst size were calculated from the plotted curve visualized in SigmaPlot v.12.5 with error bars representing standard deviation for three replicates.

### Phage immunity test

The potentially phage-immune cells of *B. cereus* VKM B-370 were collected from the central part of five turbid plaques formed by the Sam46-T phage and then plated onto LB agar plates by the streak plate method and incubated overnight at 37 $^\circ$C. Single colonies were transferred into liquid LB medium and grown overnight at 37 $^\circ$C with shaking. Next, 30 $\upmu$l of the overnight bacterial cultures were transferred into 3 ml of fresh LB medium and incubated with shaking at 37 $^\circ$C until OD590 reached 0.4–0.45. The mid-log phase bacterial cultures were diluted to the OD590 of 0.2. Fifty microliters of the Sam46-T and Sam46-C phages at the concentrations of $6\times 10^7$ PFU/ml were added separately into a 48-well microplate to 450 $\upmu$l of diluted bacterial cultures to provide MOI of about 2. The microplate was incubated for 7 h at 30 $^\circ$C in the FilterMax F5 microplate reader (Molecular Devices), with OD595 being measured every 10 min. Non-infected cultures were used as control samples. Optical density was measured three times for both original *B. cereus* VKM B-370 cells and the potentially phage-immune *B. cereus* VKM B-370 cells. The obtained growth curves were visualized in SigmaPlot 12.5 with error bars representing standard deviation for three replicates.

### Accession number

The genome sequences and associated data for phages Sam46 and Sam112 were deposited under GenBank accession number MN604698.1 and MN604230.1, BioProject accession numbers PRJNA686639 and PRJNA686641, BioSample accession numbers SAMN17119830 and SAMN17119843, SRA accession numbers SRR13276100 and SRR13276285, respectively. Associated data for the T-type and C-type Sam46 phage genomes were submitted under BioProject accession number PRJNA686639, BioSample accession numbers SAMN17119841 and SAMN17119842, SRA accession numbers SRR13276014 and SRR13276013, respectively.

## Results

### Isolation, host range and morphology

*Bacillus* phages Sam46 and Sam112 were isolated from soil samples collected in Samara, Russian Federation. Both Sam46 and Sam112 produced clear and turbid plaques, approximately 1 mm in diameter, on the lawn of the host strain VKM B-370 (Supplementary Information, Fig. S1 and Fig. S2). Purified phage suspensions of both Sam46 and Sam112 that stably produced only one type of plaques were obtained after five cycles of the extraction-titration procedure. Thus, four phages were ultimately obtained: Sam46-C, Sam46-T, Sam112-C and Sam112-T. The letters C and T indicate the morphotypes of plaques formed by the phages: clear and turbid morphotype, respectively. A host range test was carried out for 32 strains of the *B. cereus* group. Both original and purified (producing uniform plaques) suspensions of Sam46 and Sam112 were capable of forming plaques on the lawns of 17 and 16 (out of 32) strains ($\sim$ 50%) , respectively (Table 1).

**Table 1:** The host range of *Bacillus* phages Sam46 and Sam112 for 32 *Bacillus* strains.

No.	Species	Strain	Sam46 (O*)	Sam46 (C**)	Sam46 (T***)	Sam112 (O*)	Sam112 (C**)	Sam112 (T***)
1	*B. cereus*	VKM B-13	+	+	+	+	+	+
2	*B. cereus*	VKM B-15	−	−	−	−	−	−
3	*B. cereus*	VKM B-370	+	+	+	+	+	+
4	*B. cereus*	VKM B-373	+	+	+	+	+	+
5	*B. cereus*	VKM B-383	+	+	+	+	+	+
6	*B. cereus*	VKM B-445	−	−	−	−	−	−
7	*B. cereus*	VKM B-473	+	+	+	+	+	+
8	*B. cereus*	VKM B-491	+	+	+	+	+	+
9	*B. cereus*	VKM B-504^T^	−	−	−	−	−	−
10	*B. cereus*	VKM B-682	−	−	−	−	−	−
11	*B. cereus*	VKM B-683	+	+	+	+	+	+
12	*B. cereus*	VKM B-684	+	+	+	−	−	−
13	*B. cereus*	VKM B-686	+	+	+	+	+	+
14	*B. cereus*	VKM B-688	+	+	+	+	+	+
15	*B. cereus*	VKM B-771	−	−	−	−	−	−
16	*B. cereus*	VKM B-810	−	−	−	−	−	−
17	*B. cereus*	VKM B-812	−	−	−	−	−	−
18	*B. cereus*	VKM B-861	+	+	+	+	+	+
19	*B. cereus*	ATCC 4342	−	−	−	−	−	−
20	*B. cereus*	ATCC 14893	−	−	−	−	−	−
21	*B. thuringiensis*	VKM B-83	−	−	−	−	−	−
22	*B. thuringiensis*	VKM B-84	−	−	−	−	−	−
23	*B. thuringiensis*	VKM B-440	+	+	+	+	+	+
24	*B. thuringiensis*	VKM B-443	−	−	−	−	−	−
25	*B. thuringiensis*	VKM B-446	+	+	+	+	+	+
26	*B. thuringiensis*	VKM B-447	−	−	−	−	−	−
27	*B. thuringiensis*	VKM B-450	+	+	+	+	+	+
28	*B. thuringiensis*	VKM B-454	+	+	+	+	+	+
29	*B. thuringiensis*	VKM B-1555	+	+	+	+	+	+
30	*B. thuringiensis*	VKM B-1557	+	+	+	+	+	+
31	*B. thuringiensis*	ATCC 35646	−	−	−	−	−	−
32	*B. weihenstephanensis*	KBAB4	−	−	−	−	−	−

O*, original phage suspension producing two types of plaques: clear and turbid; C** and T***, purified phage suspensions producing only one type of plaques: clear plaques or turbid plaques, respectively.

**Figure 1:**
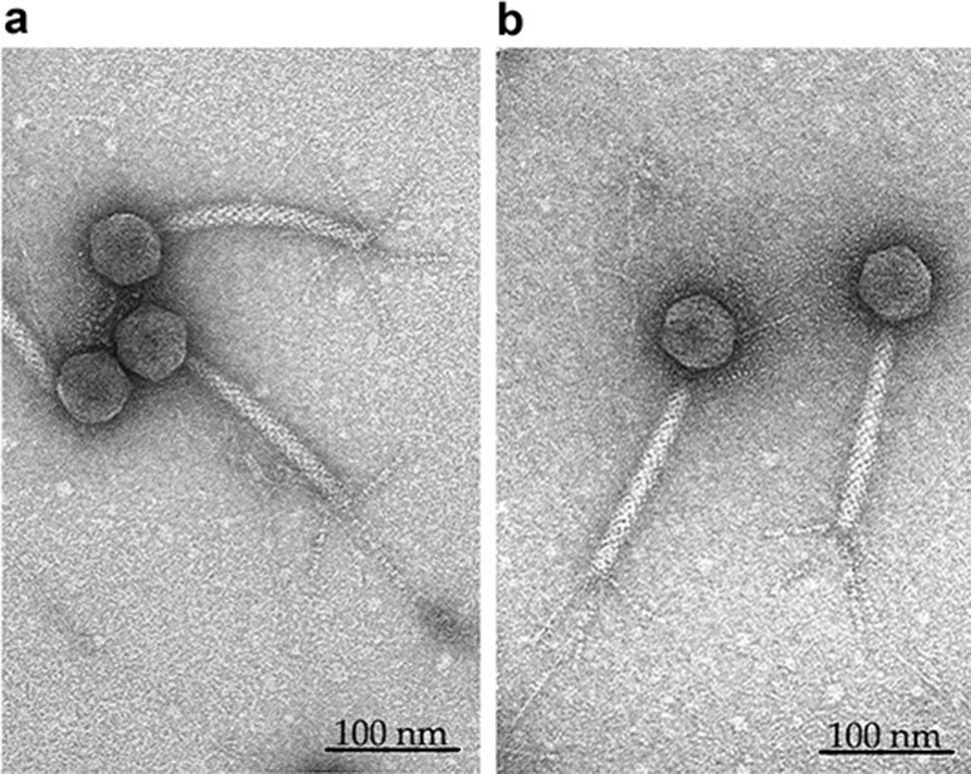
Transmission electron microscopy of *Bacillus* phages (**a**) Sam46 and (**b**) Sam112 negatively stained with 1% (w/v) uranyl acetate. Scale bar 100 nm. The full-length TEM micrographs of Sam46 and Sam112 are presented in Supplementary Figures S3 and S4, respectively.

The TEM analysis revealed that both Sam46 and Sam112 (for each phage, n = 10 phage particles) possess an icosahedral non-elongated head of approximately $\ 57.62 \pm 1.2$ nm in diameter attached to a characteristic long contractile nonflexible tail of approximately $\ 160.0 \pm 5.5$ nm in length (including the baseplate structure) and $\ 16.9 \pm 1.3$ nm in width, ending with a baseplate structure with six tail fibers. These phages have the characteristic morphological features of the myovirus morphotype (Fig. 1).

### General genome organization of Sam46 and Sam112

**Figure 2:**
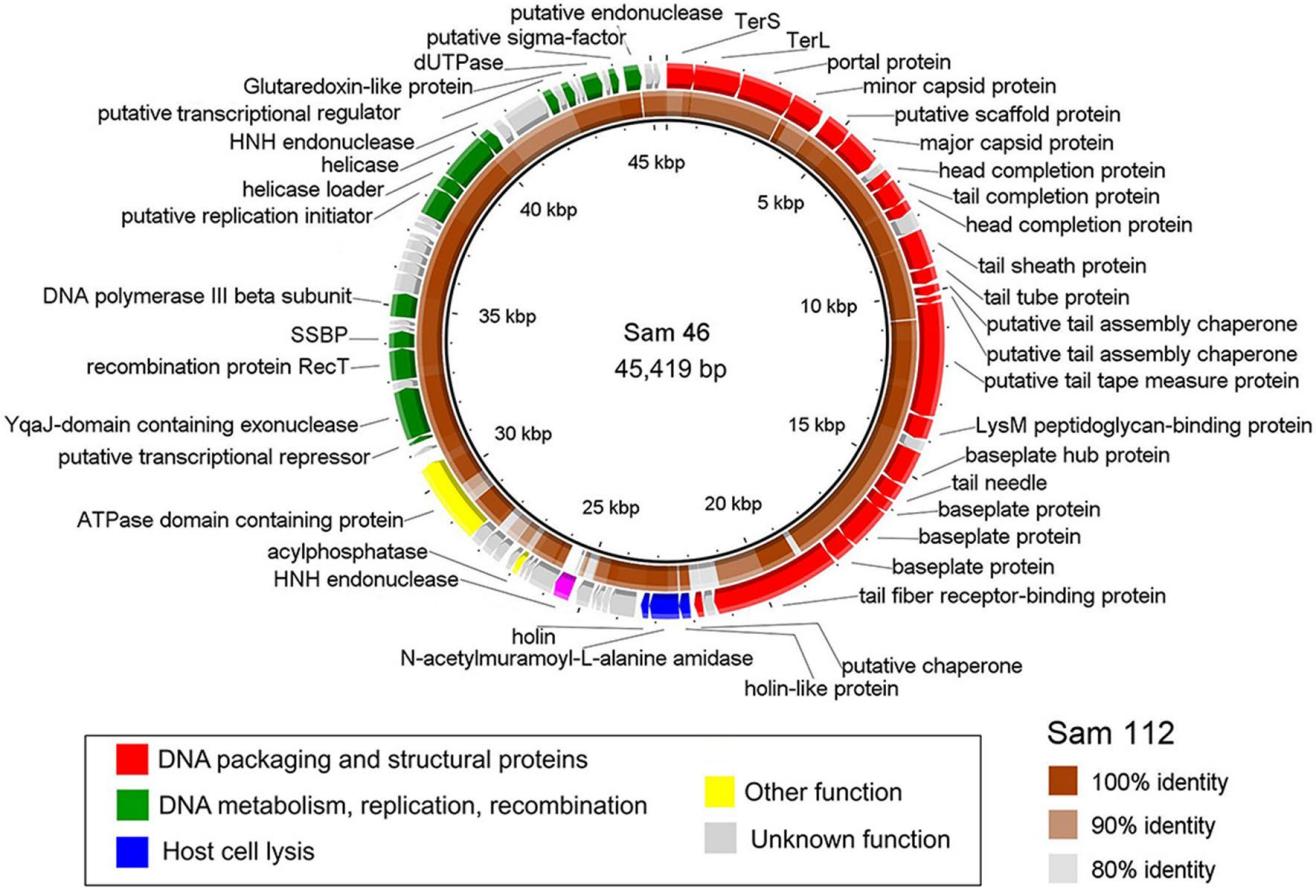
The Sam46 genome map visualized with BRIG software v.0.95 (http://brig.sourceforge.net). The inner ring shows the level of Sam112 genomic identity (tBLASTx e-value significance) to Sam46, from unrelated (white regions) to closely related (dark brown), according to the legend. Fuchsia arrow in Sam46 genome indicates the HNH-endonuclease absent in Sam112.

The Sam46 genomic sequence contains 45,419 bp with the GC-content of 41.6% and 77 predicted ORFs. The Sam112 genome is slightly shorter, with 45,037 bp, 75 predicted ORFs and the same GC-content. According to the BLASTn (NCBI), these phages have the whole genome identity value of 96.0% (Supplementary Information, Table S3), indicating that they belong to the same phage species in accordance with the official ICTV classification, as the currently used species demarcation criterion is the genome nucleotide identity of 95%^53,54^. The circular genome map of Sam46 is shown in Fig. 2, with the first base of small terminase subunit gene selected as the starting point of the genome.

The putative functions were assigned to 44 (57.1%) and 44 (58.7%) ORFs of Sam46 and Sam112, respectively, using BLASTp (NCBI)^33^ and HHpred^34^. More detailed information about the predicted ORFs is provided in Tables S1 and S2 (Supplementary Information, Table S1, Table S2).

#### DNA packaging genes

In the case of both phages Sam46 and Sam112, ORF1 (locus_tag: Sam46_gp1, Sam112_gp1) and ORF2 (locus_tag: Sam46_gp2, Sam112_gp2) encode the small and large terminase subunit, respectively. ORF1s have been found to possess a two-domain structure: C-terminal Terminase_2 domain (residues 107–230) (E-value $1.2e^{-27}$), the typical domain of phage small terminase subunits, and N-terminal FtsK_gamma domain (residues 11–70) (E-value $\sim 2e^{-27}$), which is occasionally encoded in a separate ORF in some phages but not in such a fusion. The role of single FtsK_gamma-like genes in phage genomes is still unknown. The FtsK_gamma domain is a winged helix subdomain of the C-terminal DNA translocation motor component of FtsK and SpoIIIE proteins, coordinating the proper segregation of bacterial chromosomes during cell division and sporulation, respectively^55,56^. The bacterial FtsK_gamma domain acts as a DNA-binding domain that recognizes and binds the specific chromosomal sequences of 8 bp in length, known as KOPS (FtsK Orienting Polarized Sequences)^55,57,58^ or FRS (FtsK Recognition Sequence), and in the case of SpoIIIE known as SRS (SpoIIIE Recognition Sequence)^59^.

**Figure 3:**
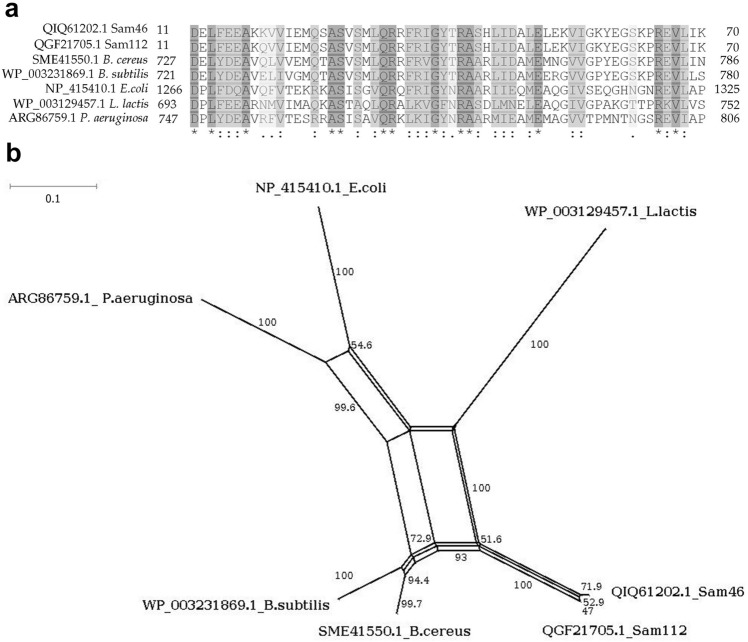
The sequence analysis of FtsK-like protein gamma domain. (**a**) Sequence alignment of the FtsK-like protein gamma domain by MAFFT version 7 (https://mafft.cbrc.jp/alignment/server). Accession numbers and species name are labeled on the left side of each alignment. Numbers located on both sides of the alignments indicate the exact positions of each sequence. (**b**) Neighbor-Net network tree of FtsK_gamma domains by SplitTree4 v.4.16.1 (https://software-ab.informatik.uni-tuebingen.de/download/splitstree/welcome.html). Accession number and species name are labeled at the ends of the branches. Numbers on the branches indicate bootstrap test values with 1000 replicates. Scale bar: number of substitutions per site.

The alignment of the FtsK_gamma domains of Sam46 and Sam112 small terminase subunits with the bacterial FtsK_gamma domains (*B. cereus*, *B. subtilis*, *E. coli*, *L. lactis* and *P. aeruginosa*) (Fig. 3a) has revealed that the DNA binding sites of FtsK_gamma domains of both phages are highly conserved among bacteria. The phylogenetic analysis of FtsK_gamma domains indicated that those from *B. cereus* and *B. subtilis* are most closely related to the FtsK_gamma domains of Sam46 and Sam112 (Fig. 3b). Therefore, the FtsK_gamma domains of the small terminase subunits of Sam46 and Sam112 were probably acquired from bacterial hosts during co-evolution.

Searching Sam46 and Sam112 genomes for bacterial KOPS/SRS such as *B. subtilis*: 5$'$-GAGAAGGG-3$'$^59^; *E. coli*: 5$'$-GGGNAGGG-3$'$^57^,*L. lactis*: 5$'$-GAGAAG-3$'$^58^ and *P. aeruginosa* 5$'$-GGGCAGGG-3$'$^60^ sequences has revealed the presence of only two *E. coli* KOPS and 15 *L. lactis* KOPS for both phages.

Atypical domain structure of the small terminase subunits of phages Sam46 and Sam112 indicates that the Sam46 and Sam112 genomes might contain KOPS/SRS-like binding sites for the small terminase.

#### Morphogenesis and lytic genes

The morphogenesis genes of Sam46 and Sam112 were identified using BLAST and HHpred, taking into account the predicted structural similarities with the known orthologous proteins. The capsid genes with the predicted functions include SPP1 Gp6-like portal protein (Sam46_gp3, Sam112_gp3), minor capsid protein (Sam46_gp4; Sam112_gp4), putative scaffold protein (Sam46_gp5, Sam112_gp5), SPP1 Gp13-like major capsid protein (Sam46_gp6, Sam112_gp6), SPP1 Gp15-like head completion protein (Sam46_gp8, Sam112_gp8) and XkdH-like head completion protein (Sam46_gp10, Sam112_gp10). Functionally assigned tail genes include tail completion protein (Sam46_gp9, Sam112_gp9), tail sheath protein (Sam46_gp12, Sam112_gp12), tail tube protein (Sam46_gp13; Sam112_gp13), putative tail tape measure protein (Sam46_gp16; Sam112_gp16), LysM domain-containing peptidoglycan-binding protein (Sam46_gp17, Sam112_gp17), baseplate hub protein (Sam46_gp19, Sam112_gp19), tail needle (Sam46_gp20, Sam112_gp20), three baseplate proteins (Sam46_gp21, Sam112_gp21; Sam46_gp22, Sam112_gp22; Sam46_gp23, Sam112_gp23, respectively) with predicted structural homology to Gp25 T4, GpJ and GpI P2, tail fiber receptor-binding protein (Sam46_gp24, Sam112_gp24) and three putative tail assembly chaperones (Sam46_gp14, Sam112_gp14; Sam46_gp15, Sam112_gp15; Sam46_gp16, Sam112_gp16, respectively).

Although Sam46 and Sam112 share several structural genes with bacteriophage SPP1, a member of the *Siphoviridae* family, their structural gene modules are much more similar to that of the deep-sea thermophilic phage D6E (Fig. 6), a myovirus with a highly mosaic genome^61^.

#### Replication and recombination genes

The module of replication- and recombination-related genes of Sam46 resembles that of the virulent *Bacillus* bacteriophage SPP1 (Fig. 2) and belongs to the “initiator-helicase-helicase loader” type according to the classification proposed by Weigel and Seitz^62^. It includes the adjacently encoded DnaD domain-containing putative replication initiator (Sam46_gp61; Sam112_gp58), lambda P-related helicase loader (Sam46_gp62; Sam112_gp59) and DnaB-type replicative helicase (Sam46_gp63; Sam112_gp60). Sam46_gp47 (Sam112_gp60) and Sam46_gp49 (Sam112_gp47) encode products with apparent homology to YqaJ domain-containing exonuclease and RecT-like protein, respectively, which thereby seem to be parts of the two-component recombination system functionally similar to SPP1 Gp34.1^63^ and Gp35^64^. Sam46_gp46 (Sam112_gp48) is an *E. coli*-type single stranded DNA binding (SSB) protein known to be frequently associated with such a recombination system^62,65^. Sam46_gp53 (Sam112_gp50) is the processivity factor (DNA sliding clamp) of PolIII$\beta$-type, which is most abundant among bacteriophages^62^.

### Comparative genomics

**Figure 4:**
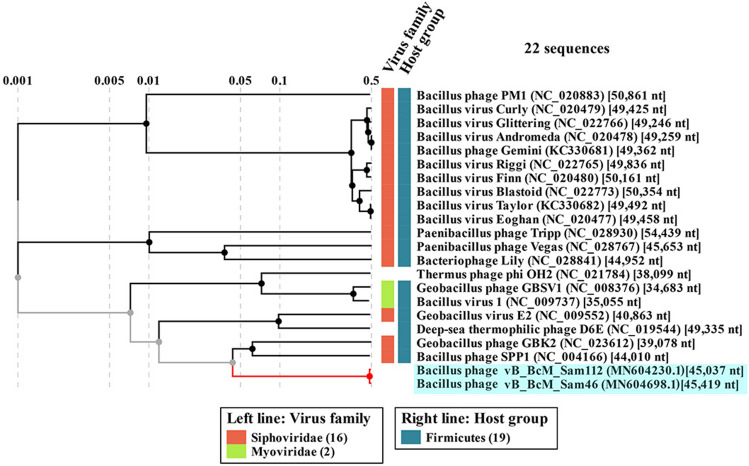
Phylogenetic relationship of Sam46 and Sam112 to the known phages. The tree was generated using the ViPTree server version 1.9 (https://www.genome.jp/viptree/) based on genome-wide sequence similarities computed by tBLASTx. The clade containing the members of of the proposed new genus ‘Samaravirus’ is highlighted in light blue.

To assess the phylogenetic relationship of the Sam46 and Sam112 to known phages, ViPTree server version 1.9 was used to generate a proteomic tree based on the genome-wide sequence similarities computed by tBLASTx (Fig. 4). As can be seen in Figure 4, Sam46 and Sam112 form a separate branch significantly distant from the closest relatives. The most closely related genomes belong to bacteriophages SPP1 and GBK2, which are both siphoviruses.

**Figure 5:**
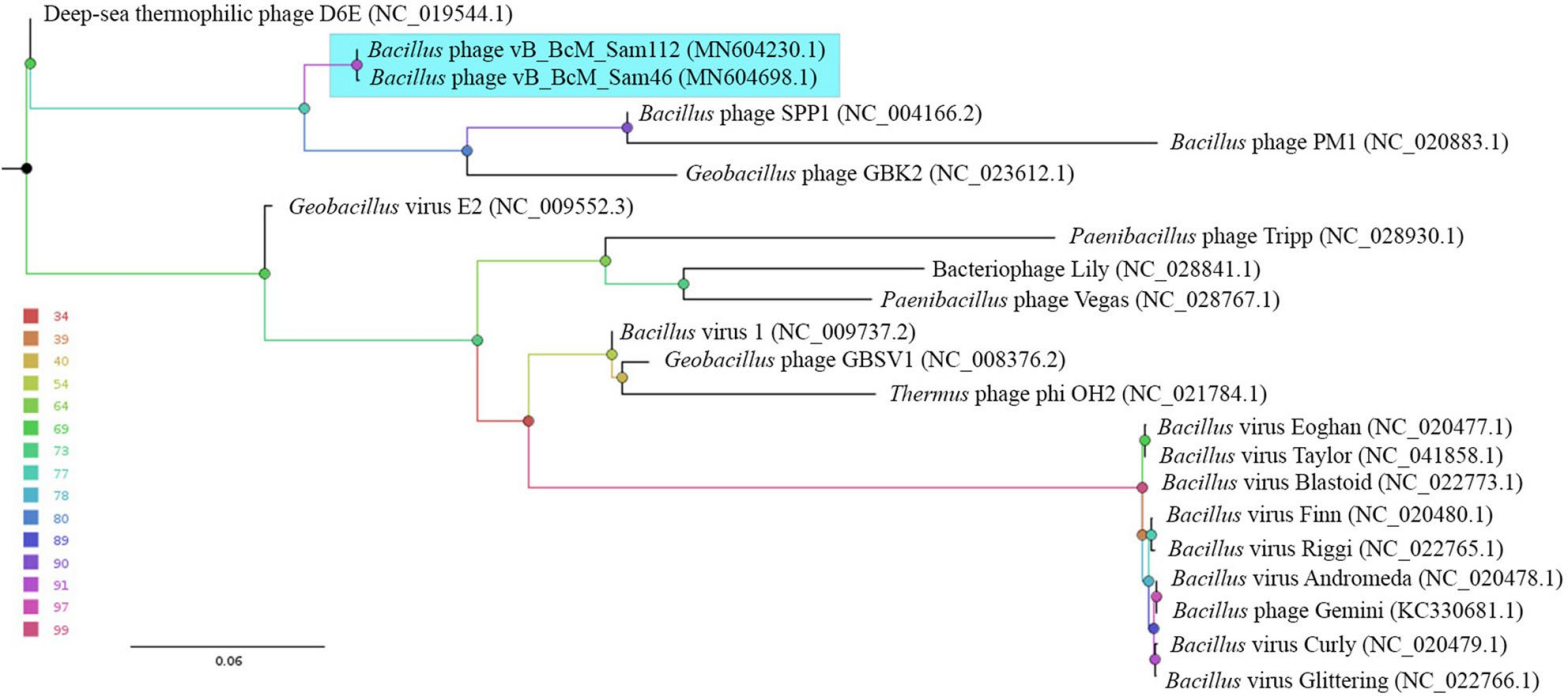
The Maximum-likelihood pan-genome tree for Sam46, Sam112 and the closest viruses inferred from the presence/absence pangenome matrix using GET_PHYLOMARKERS software package version 2.2.8.1 (http://github.com/vinuesa/get_phylomarkers) and visualized with FigTree v1.4.4 (http://tree.bio.ed.ac.uk/software/figtree/). The nodes are colored according to the legend, which represents standard bootstrap support values. The clade containing the members of the proposed new genus ‘Samaravirus’ is highlighted in blue.

In addition, the number of shared proteins was computed using the GET_HOMOLOGUES software (Supplementary Information, Table S4), and a pangenome matrix was produced showing the presence/absence of representatives of individual gene/protein clusters in each genome. Based on the matrix, a Maximum-likelihood (ML) tree was drawn (Fig. 5), where the intergenomic distances are computed from the number of shared proteins. According to Table S3 and Fig. 5, the myovirus D6E shares slightly more proteins (20) than SPP1 (16) with Sam46. Thus, the VipTree and GET_PHYLOMARKERS phylogenetic analyses failed to give consistent results, which is not in any way surprising: both SPP1 and D6E share less than 23% of their proteins with Sam46, therefore, for the question which one of them is the closest relative to make sense, both are far too distant.

**Figure 6:**
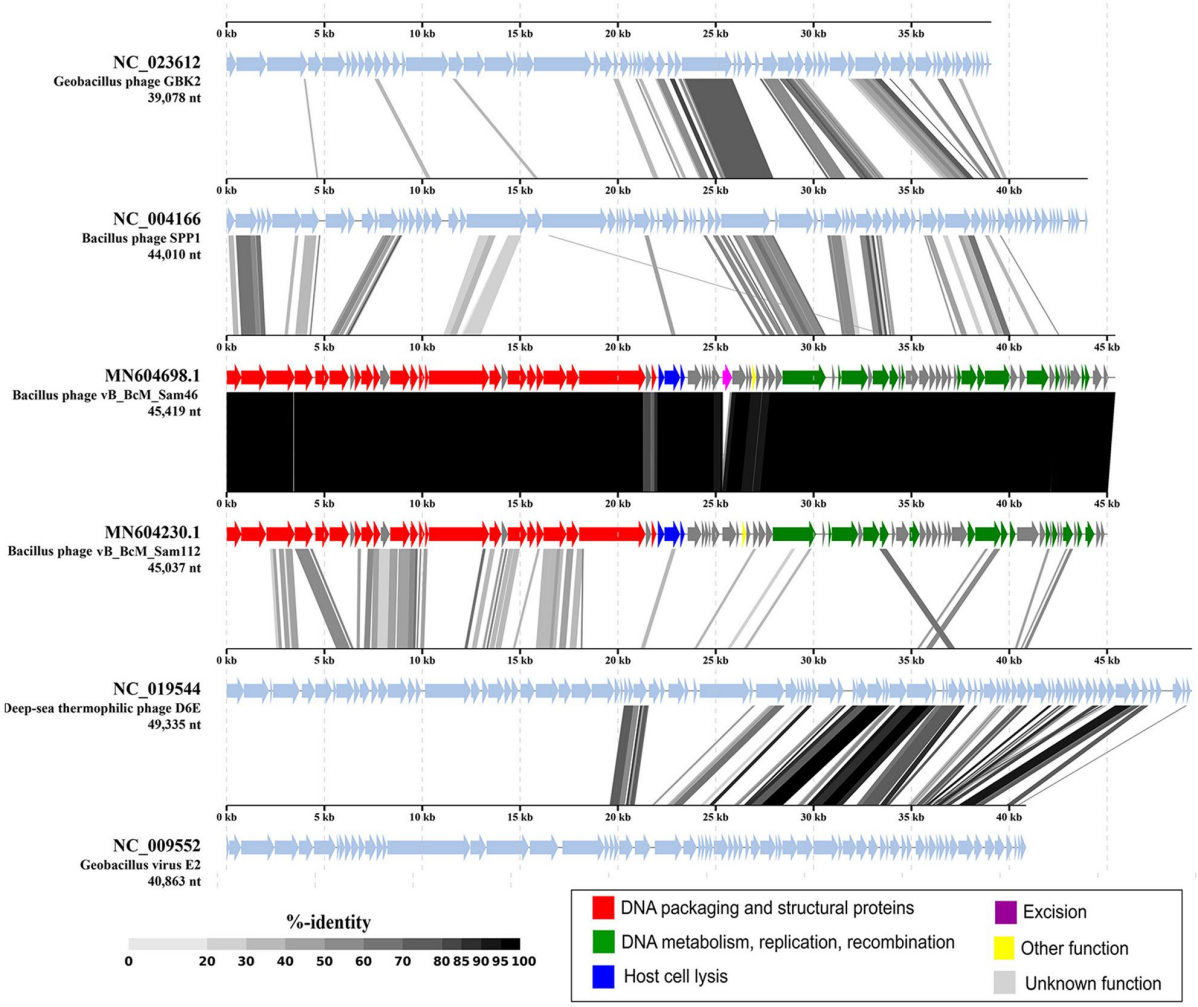
The tBLASTx comparison of the genomes of Sam46, Sam112 and the most related phages visualized with ViPTree server version 1.9 (https://www.genome.jp/viptree/). For Sam46 and Sam112, the color scheme corresponds to the supposed functions (the same as in Fig. 2). Fuchsia arrow in Sam46 genome indicates the HNH-endonuclease absent in Sam112.

The linear whole genome comparison diagram was also visualized with the ViPTree server version 1.9 (Fig. 6), showing tBLASTx pairwise similarities between the most closely related genomes. As illustrated on the diagram, both the SPP1 and D6E genomes contain regions of local similarity to the Sam46 and Sam112 genomes, although within different gene modules. D6E shares many structural genes with Sam112, whereas the similarity between SPP1 and Sam46 is mostly limited to replication-related genes. This indicates that different parts of the Sam46 and Sam112 genomes were acquired from different sources and that phages that are more closely related to Sam46 and Sam112 are still to be discovered. The discovery of such phages would allow us to reconstruct the history of the biggest recombination events that have led to the observed mosaicity of the Sam46 and Sam112 genomes.

### Determination of packaging strategy

**Figure 7:**
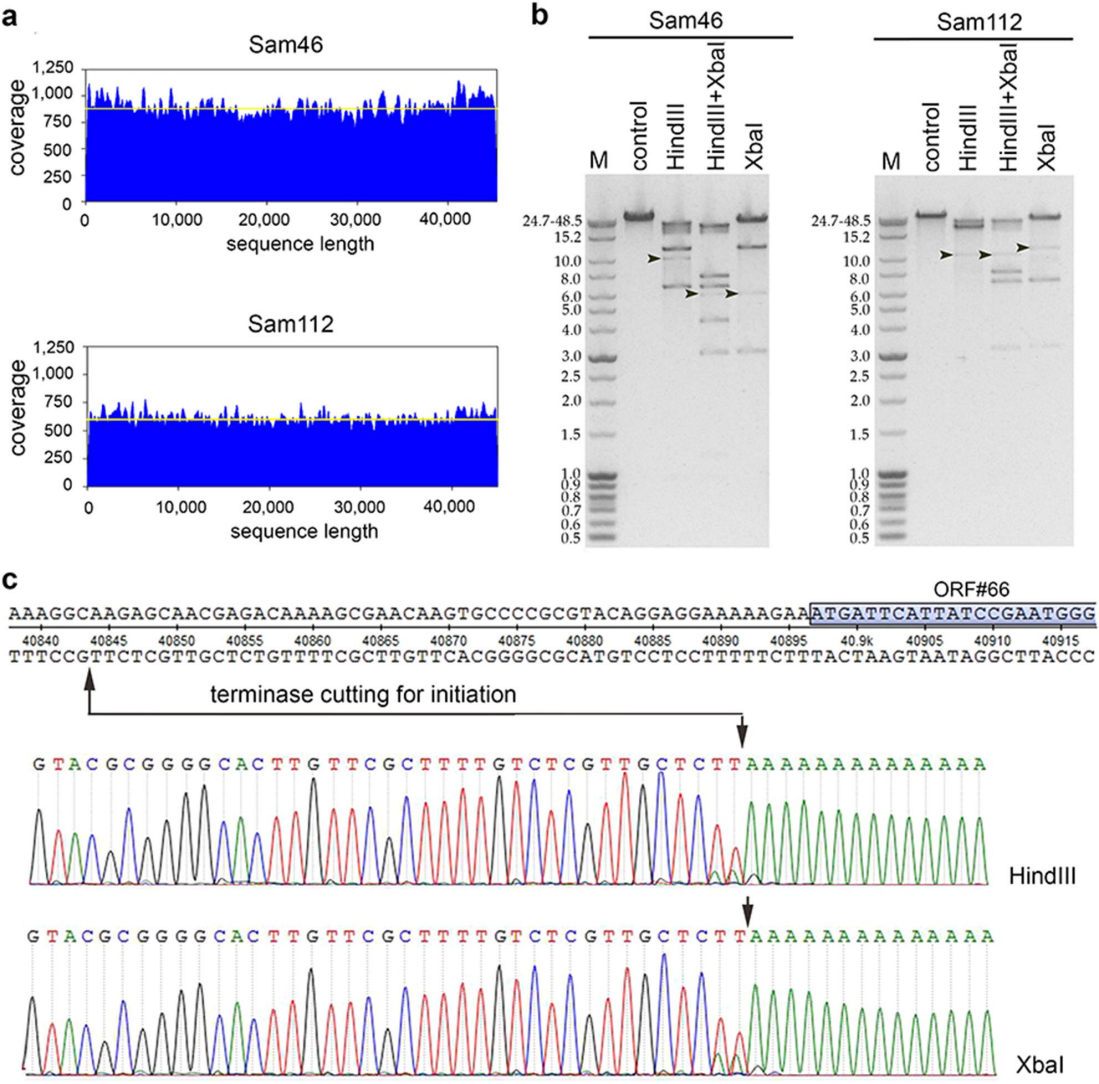
Determination of packaging strategy. (**a**) Genome coverage plot was implemented with the Bowtie2 software tool v.2.3.4.3 (https://cpt.tamu.edu/galaxy-pub). No peaks deviate significantly from the mean (yellow line). (**b**) Restriction analysis of Sam46 and Sam112 DNA with enzymes XbaI and HindIII; M—molecular weight markers; black arrows indicate *pac*-fragments. The full-length gel is presented in Supplementary Figure S5. (**c**) Location of the terminase-generated cut in the Sam46 genome. The terminal regions of the PCR product sequences obtained with RAGE for HindIII- and XbaI-generated *pac*-fragments are shown on the sequencing chromatograms.

In the case of both Sam46 and Sam112, relatively homogeneous coverage with no significant peaks (with double or more than double depth of coverage) was observed on the coverage plot by the Bowtie2 software tool v.2.3.4.3 (Fig. 7a), which is typical of phages with circularly permuted terminal repeats using the headful mechanism of DNA packaging^37^. The DNA packaging mode of Sam46 and Sam112 was experimentally confirmed by restriction analysis of the genomic DNA using restriction endonucleases HindIII, XbaI and HindIII with XbaI simultaneously. For both Sam46 and Sam112, on each track of the electrophoregrams we could find all of the fragments predicted in silico from artificially circularized genomic sequences and also an additional unexpected fragment appearing in submolar concentrations relative to others (Fig. 7b)^66^. This type of restriction pattern is known to be characteristic of phages with the headful mode of DNA packaging^66^. The additional submolar fragments are so-called “*pac*-fragments”, which are produced during the first packaging event at each genomic concatemer and, unlike true restriction fragments, are cut on one side by the phage terminase. The observed lengths of the *pac*-fragments allowed us to predict the approximate location of the *pac*-site, which is expected to be roughly 4.5 Kbp upstream of the TerS gene in both Sam46 and Sam112 genomes.

The Sam46 *pac*-fragments were extracted from the electrophoresis gel and used for determination of the *pac*-site location using the RAGE method followed by Sanger sequencing of the products obtained in the second PCR. For the HindIII- and XbaI-generated *pac*-fragments, the terminase-generated cut was found to be approximately at position 40,843 in the intergenic region upstream of Sam46_gp66, as is shown on the sequencing chromatograms (Fig. 7c). Thus, both Sam 46 and Sam112 apparently use the headful mechanism of DNA packaging with accurate site-specific initiation.

### Identification of genetic differences between the phages with turbid and clear plaque morphotypes

**Figure 8:**
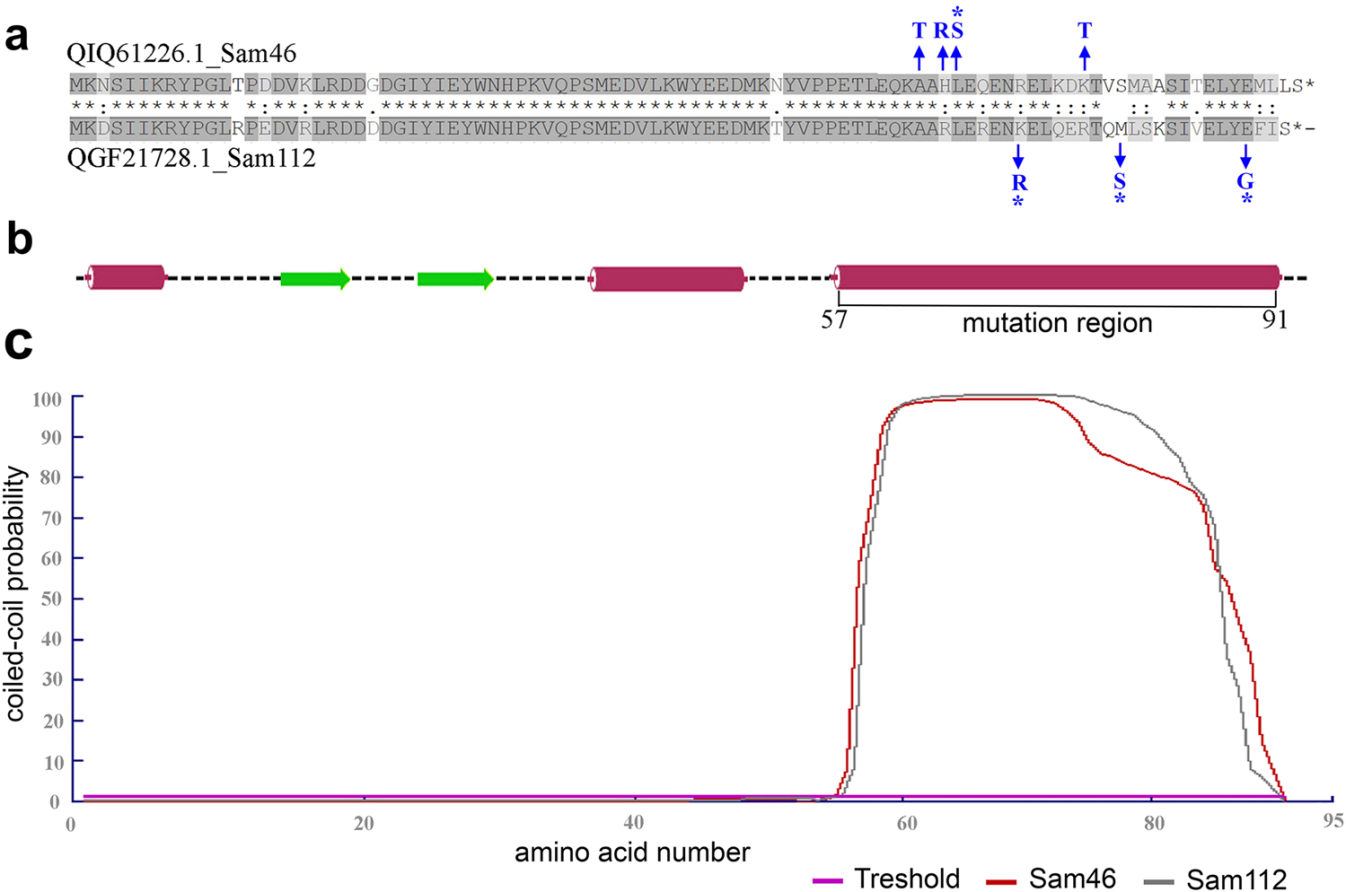
The sequence analysis of XkdW-like protein. (**a**) Sequence alignment of XkdW-like proteins of phage Sam46 and Sam112 by MAFFT version 7 (https://mafft.cbrc.jp/alignment/server). Accession numbers are labeled for each sequence. The mutations obtained from WGS data analysis are indicated in blue and the mutations found in Sanger sequencing data are indicated in blue with asterisks. (**b**) Secondary structure prediction for the XkdW-like protein of Sam46 by JPRED4 (http://www.compbio.dundee.ac.uk/jpred/) ($\beta$-sheet: green arrow; $\alpha$-helix: burgundy block). C-terminal $\alpha$-helix is the mutation region. (**c**) Coiled-coil probability plotted versus the amino acid sequence of the XkdW-like protein of Sam46 and Sam112. The probabilities were determined using the LOGICOIL server (http://coiledcoils.chm.bris.ac.uk/LOGICOIL/).

DNA was extracted from the Sam46-T and Sam46-C phages as described in “Materials and methods”. The whole genome sequencing of the Sam46-T and Sam46-C genomes showed differences in the genetic content of only one gene. The Sam46-T genome is completely identical to the originally sequenced Sam46 genome, while the Sam46-C genome contains mutations in the gp25 gene (gene locus_tag: Sam46_gp25; protein ID: QIQ61226.1) encoding XkdW-like protein with unknown function (Fig. 8a).

The series of experiments on obtaining mutant phages with clear plaque morphotype Sam46 and Sam112 from Sam46-T and Sam112-T, respectively, followed by Sanger sequencing of the gp25 gene, confirmed the appearance of point mutations within this gene in C-type phages (Fig. 8a). The frequency of turbid-to-clear plaque mutations in both Sam46 and Sam112 was 1:3000.

All mutations identified in both WGS and the experiment on obtaining mutant C-type phages were found to be located strictly in the C-terminal region of the XkdW-like protein (Fig. 8a,b). Secondary structure prediction by JPRED4^49^ and coiled-coil region prediction by LOGICOIL^50^ have shown that the mutation region is an $\alpha$-helix containing HPPHPPP patterns, referred to as heptad repeats (abcdfg)^67^, where hydrophobic residues (H) generally occupy the “a” and “d” positions, and polar residues (P) occur on other positions (Fig. 8b,c). Such a structure of $\alpha$-helix is characteristic of the coiled-coil motif in proteins. According the LOGICOIL prediction^50^, the most probable state of coiled coils of XkdW-like proteins of both Sam46 and Sam112 is a parallel dimer.

The experiments on obtaining mutant phages with the turbid plaque morphotype from Sam46-C and Sam112-C showed no results. Perhaps, the probability of the clear-to-turbid transition is extremely low.

### Thermal and pH stability of the Sam46-T and Sam46-C phages

Taking into account the fact that the Sam46 and Sam112 phages are the same species according to the genome-wide analysis (the whole genome identity of 96.0%), this and the subsequent experiments were performed only with Sam46 as a representative of this species. In an attempt to explain the origin of two types of plaques, we performed the experiment for both Sam46-T and Sam46-C phages.

**Figure 9:**
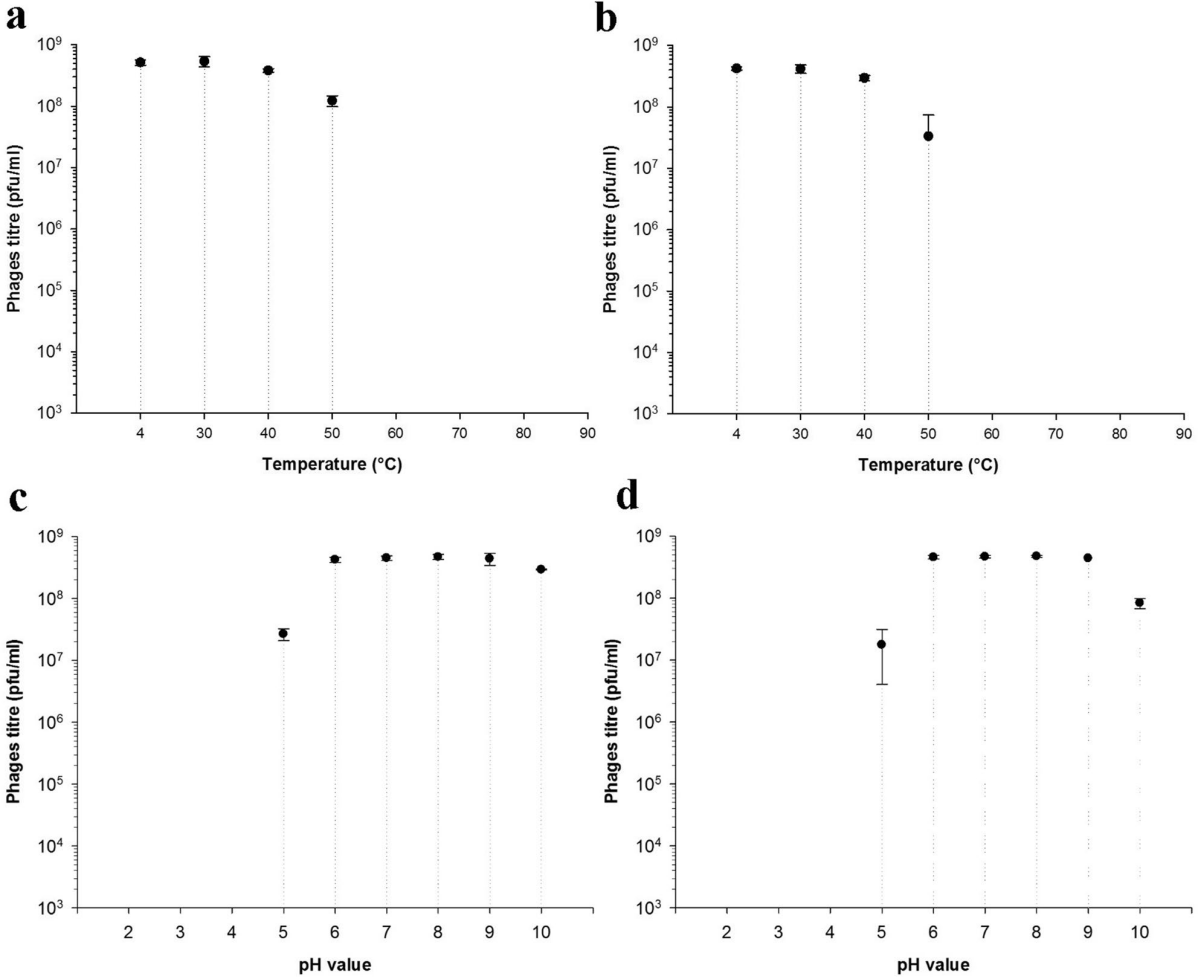
Thermal stability of the (**a**) Sam46-T and (**b**) Sam46-C phages. pH stability of the (**c**) Sam46-T and (**d**) Sam46-C phages. The graphs was created with SigmaPlot v.12.5 (http://www.sigmaplot.co.uk/products/sigmaplot/produpdates/prod-updates18.php). Error bars represent standard deviation for three replicates.

Both Sam46-T and Sam46-C phages were stable at temperatures of 30, 40 and 50 $^\circ$C, as phage titers were similar to those of the control samples incubated at 4 $^\circ$C (Fig. 9a,b). At 60 $^\circ$C or higher, the phage titer dropped sharply, no viral particles were detected after 1 h of incubation, and phage activity was completely lost (Fig. 9a,b). In addition, the results of pH stability tests showed that both Sam46-T and Sam46-C phages were stable at pH values from 5 to 10 (Fig. 9c,d). However, the Sam46-T and Sam46-C phages are not able to survive under highly acidic conditions, as no phages survived incubation in solutions at pH 2.2, 3 and 4.

### Killing assay

**Figure 10:**
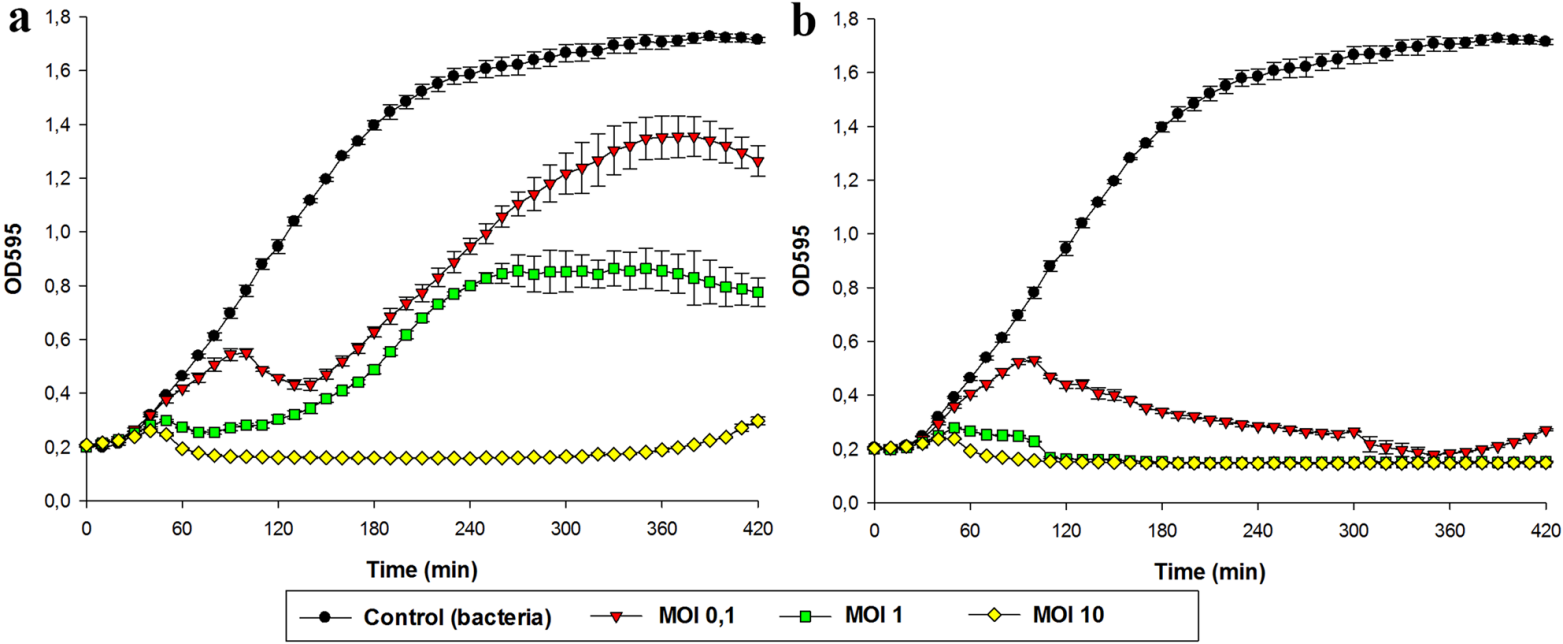
*B. cereus* VKM B-370 growth kinetics upon the infection with the (**a**) Sam46-T and (**b**) Sam46-C phages with different multiplicity of infection. Non-infected *B. cereus* VKM B-370 culture was used as a control. The graphs was created with SigmaPlot v.12.5 (http://www.sigmaplot.co.uk/products/sigmaplot/produpdates/prod-updates18.php). Error bars represent standard deviation of the means for three replicates.

*Bacillus cereus* VKM B-370 growth kinetics upon Sam46-C and Sam46-T infections at different MOI was studied by measuring OD595 of the cultures infected with C-type or T-type phages and comparing it to that of the non-infected control culture. The resultant bacterial growth curves are shown in Fig. 10. Both Sam46-T and Sam46-C infections resulted in the inhibition of bacterial growth, which became stronger as the MOI increased. However, the *B. cereus* VKM B-370 growth patterns upon Sam46-C and Sam46-T infections differ significantly at the MOI of 0.1 and 1. The observed difference in the rates of host cell lysis, and, consequently, in the plaque morphotypes of Sam46-C and Sam46-T (Supplementary Information, Fig. S1) may have different explanations, the simplest one being the formation of lysogens. However, in the ‘Phage immunity test’ section below we show that it does not appear to be the case.

### Phage immunity test

As has been mentioned above, the difference between the Sam46-C and Sam46-T plaque morphotypes (Supplementary Information, Fig. S1), as well as the difference between the rates of *B. cereus* VKM B-370 lysis (Fig. 10) upon Sam46-T and Sam46-C infection, may be related to the formation of lysogens. Temperate phages, in the prophage stage, are known to make the host bacterium resistant to viruses identical or closely related to the prophages. This phenomenon is a special case of what is known as ‘superinfection exclusion’^68,69^ and is widely used to detect the presence of prophages.

**Figure 11:**
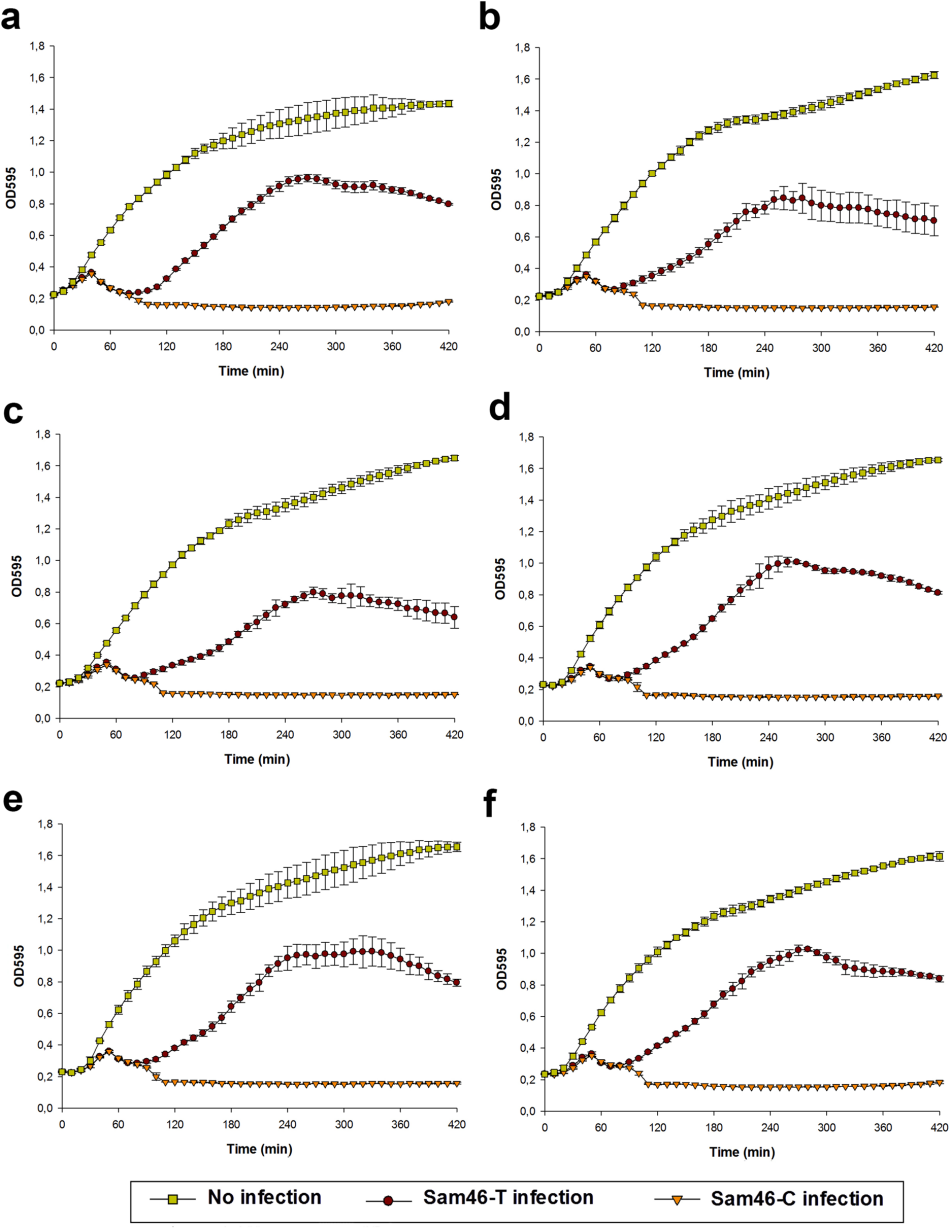
Growth kinetics of the putative phage-immune *B. cereus* VKM B-370 cultures upon the infection with the Sam46-T and Sam46-C phages. The measurement was carried out in the original *B. cereus* VKM B-370 strain used as (**a**) the control and (**b–f**) five cultures of the putative lysogenized cells collected from the central part of five separate turbid plaques formed by the Sam46-T phage. The graphs was created with SigmaPlot v.12.5 (http://www.sigmaplot.co.uk/products/sigmaplot/produpdates/prod-updates18.php). Error bars represent standard deviation of the means for three replicates.

To assess the immunity of *B. cereus* VKM B-370 cells collected from the centre of five turbid plaques (putative lysogens) against Sam46-T and Sam46-C infection, the cells were infected with Sam46-T or Sam46-C, as described in “Materials and methods”. The optical density (OD595) of the cultures was measured upon the infection, and the measurement was performed three times for both the original *B. cereus* VKM B-370 cells and the putative phage-immune cells. As is shown in Fig. 11, the optical density of the putative phage-immune *B. cereus* VKM B-370 cultures decreased similarly to that of the original *B. cereus* VKM B-370 strain.

Thus, the appearance of Sam46 and Sam112 plaques with different morphotypes and the difference in the rate of *B. cereus* VKM B-370 lysis upon T-type and C-type phage infections are not associated with the formation of lysogens.

In view of the above and considering the fact that no genes typical of temperate phages have been found in the Sam46 and Sam112 genomes (see “General genome organization of Sam46 and Sam112”), it can be concluded that the phages are virulent.

### Adsorption assay

**Figure 12:**
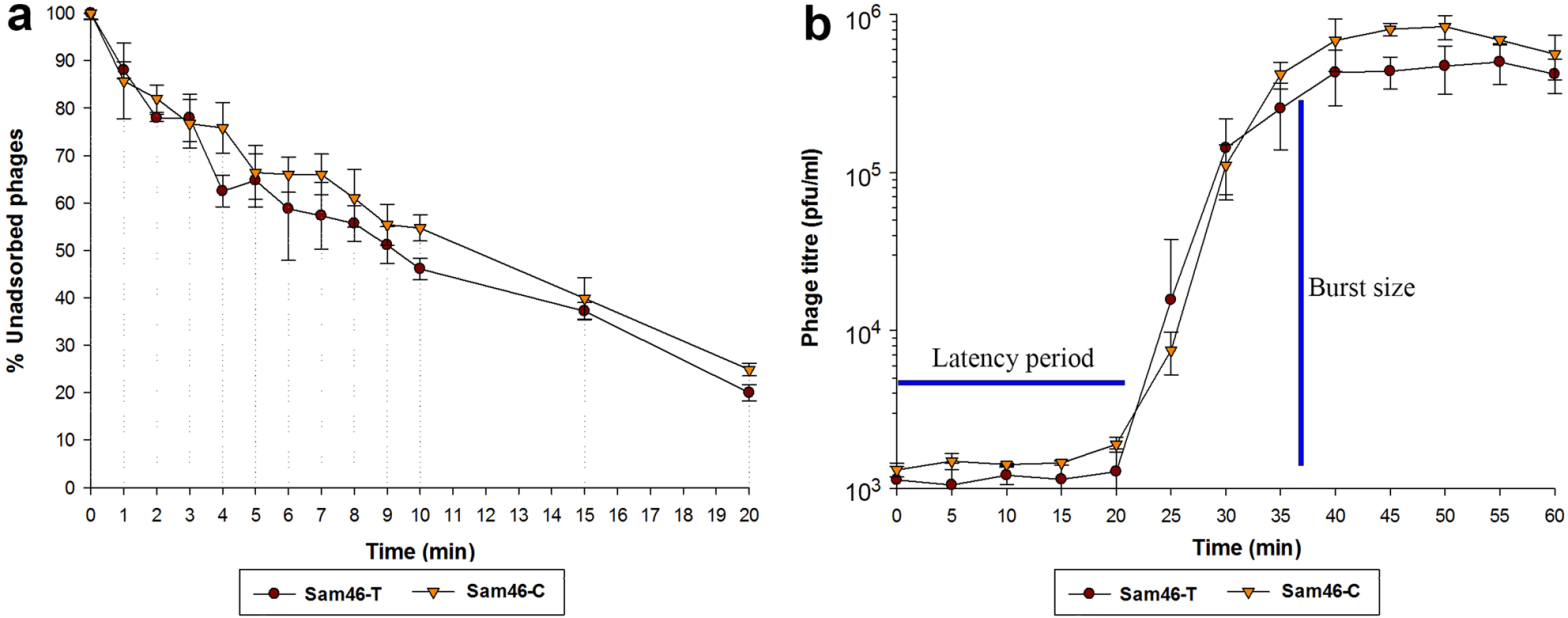
(**a**) Adsorption assay and (**b**) Growth parameters of the Sam46-T and Sam46-C phages. The graphs was created with SigmaPlot v.12.5 (http://www.sigmaplot.co.uk/products/sigmaplot/produpdates/prod-updates18.php). Error bars represent standard deviation for three replicates.

An adsorption assay was performed to identify the rate at which the Sam46-T and Sam46-C phages are adsorbed to the cell surface of *B. cereus* VKM B-370. As shown in Fig. 12, a, the adsorption rates of Sam46-T and Sam46-C are highly similar: about 50% and 80% of the phages attach to the host cells within 10 and 20 min, respectively. The adsorption rates of Sam46-T and Sam46-C are $9.68\pm 0.36\times 10^{-10}$ ml/min and $9.20\pm 0.20\times 10^{-10}$ ml/min, respectively.

### One-step growth curve

The growth kinetics of Sam46-T and Sam46-C was determined by the one-step growth curve method. The latent period of both Sam46-T and Sam46-C is about 20 min and the duration of one lytic cycle is 15-20 min. The burst size was found to be $\ 450.5 \pm 70.5$ PFU and $\ 565.6 \pm 64.6$ PFU per infected cell for Sam46-T and Sam46-C, respectively (Fig. 12b).

## Discussion

In this study, we have isolated and characterized new virulent bacteriophages Sam46 and Sam112 with a lytic activity against the *Bacillus cereus* group. According to the morphological analysis, these phages have the myovirus morphotype. The genomes of Sam46 and Sam112 contain dsDNA with the length of 45,419 bp and 45,037 bp, respectively, and the same GC-content of 41.6%. Phages Sam46 and Sam112 have a high level of the whole genome nucleotide identity (96.0%) indicating that they belong to the same phage species^53^.

Both Sam46 and Sam112 were able to form two types of plaques: clear (C-type) and turbid (T-type) on the lawn of the host strain *B. cereus* VKM B-370. The purified C-type and T-type phages were able to stably produce uniform plaques in the following generations. As has been shown, the appearance of Sam46 and Sam112 plaques with different morphotypes and the difference in the rate of *B. cereus* VKM B-370 lysis upon T-type and C-type phage infections are not associated with the formation of lysogens (Fig. 11). The genome-wide analysis of C-type and T-type Sam46 phages suggests that the phenotypic difference of plaques is due to mutations in the gp25 gene encoding the XkdW-like protein with unknown function. The structural analysis of the XkdW-like protein showed that the detected mutations were located in the coiled-coil sequence (Fig. 8). The coiled-coil motifs are found in numerous protein structures including phage structural proteins such as fibritin (gpwac) of bacteriophage T4^70,71^ and the distal subunit of the long tail fiber (gp37)^72^ of bacteriophage T4. The coiled-coil regions of such phage proteins proved to be crucial for the folding of the proteins, as well as for promoting conformational changes and contributing to protein-protein interactions^70–72^. Based on our experimental data and taking into account the gp25 gene neighboring tail-related genes (gp24, gp26), as well as considering the structural features of the XkdW-like protein it encodes, we suggest that the gp25 product is apparently involved in phage tail assembly. With this in mind, the XkdW-like protein may be related to phage adsorption or may affect characteristics of the virion assembly such as the rate of assembly and the number of active phage particles. Different adsorption rates of T-type and C-type phages would explain the phenomenon of morphologically different plaques. However, the adsorption assay did not lend credence to this hypothesis. As shown in Fig. 12 a, about 80% of the Sam46-T and Sam46-C particles are adsorbed to the host cells within 20 min, with the rates of adsorption being $9.68\pm 0.36\times 10^{-10}$ ml/min and $9.20\pm 0.20\times 10^{-10}$ ml/min, respectively. According to the results of the one-step growth curve experiment, the burst sizes of the Sam46-T and Sam46-C phages are slightly different: approximately $\ 450.5 \pm 70.5$ PFU and $\ 565.6 \pm 64.6$ PFU per infected cell, respectively. Thus, more detailed studies are needed for clear understanding of the XkdW-like protein function and its role in the Sam46 and Sam112 lifecycles.

The thermal and pH stability of Sam46-T and Sam46-C were determined. Both Sam46-T and Sam46-C phages are stable at the temperatures ranging from 4 to 50 $^\circ$C, and a further increase in temperature (60 $^\circ$C and higher) results in the complete inactivation of the phages (Fig. 9a,b). Almost 100% of both Sam46-T and Sam46-C phages survived at pH values ranging from 5 to 10, but neither phage survived at pH 2.2, 3 and 4 (Fig. 9c,d).

The predicted headful DNA packaging mechanism of Sam46 and Sam112 was experimentally confirmed by restriction analysis (Fig. 7b). We also determined the *pac*-site location using the method RAGE^48^ followed by Sanger sequencing (Fig. 7c).

An interesting feature of the Sam46 and Sam112 phages is the unusual structure of their small terminase subunit initiating DNA packaging. The small terminase subunit of these phages possesses a two-domain structure with the typical C-terminal Terminase_2 domain (residues 107–230) and N-terminal FtsK_gamma domain (residues 11–70); the latter has not been previously described as part of the small terminase subunit. Some phage genomes have been shown to contain genes encoding FtsK_gamma-like proteins, the role of which is still unclear. FtsK_gamma domain is a well-studied component of bacterial motor proteins such as the FtsK protein of *E. coli* and the SpoIIIE protein of *B. subtilis*, which are required to segregate DNA across bacterial membranes^55,59^. The FtsK_gamma domain recognizes and binds the octameric asymmetric sequences known as KOPS (FtsK Orienting Polarized Sequences)^55^ or FRS (FtsK Recognition Sequence), which in the case of SpoIIIE are known as SRS (SpoIIIE Recognition Sequence)^59^. On the one hand, such atypical domain structure of the small terminase indicates that the Sam46 and Sam112 genomes may contain KOPS/SRS-like binding sites, which act as recognizing and loading sites for the terminal complex and are likely to provide an oriented DNA translocation into procapsid. On the other hand, the FtsK_gamma domains of Sam46 and Sam112 are closely related to the FtsK_gamma domains of *B. cereus* and *B. subtilis* according to the phylogenetic analysis. This fact suggests that the small terminase can bind the SRS-sites of host DNA, resulting in the mispacking of bacterial DNA, instead of viral DNA, into their empty proheads. The majority of phages able to perform generalized transduction have been shown to package their DNA by the headful packaging mechanism^73,74^, in particular, the virulent *Bacillus* bacteriophage SPP1. The SPP1 phage is one of the closest relatives of the Sam46 and Sam112 phages according to the phylogenetic analysis (Figs. 4, 5) and is able to encapsidate chromosomal or plasmid DNA^74,75^. Generalized transduction has been well-known to require that homologous regions or pseudo-*pac* sites be presented in the host chromosomal or plasmid DNA, in order for the host DNA to be mistakenly recognized by phage terminases. Furthermore, SPP1 replication and recombination systems have recently been shown to contribute to horizontal plasmid transfer, although to a different extent^75^. We have also found that the module of replication- and recombination-related genes of Sam46 and Sam112 resembles that of SPP1 (Fig. 6). Thus, we suppose that Sam46 and Sam112 are apparently generalized transducing phages. However, the structural characteristics of the small terminase subunit of Sam46 and Sam112 and its possible role in horizontal gene transfer need further studies.

According to the phylogenetic analysis, Sam46 and Sam112 form a distinct clade located at a considerable distance from the most closely related phages, SPP1 and D6E (Figs. 4, 5). We therefore infer that Sam46 and Sam112 may be the members of a new phage genus, and we propose to create a new phage genus called ‘*Samaravirus*’ to formally classify these phages.

## Supplementary Information

Supplementary Information 1.
