# Supplementary material for: vB_BcM_Sam46 and vB_BcM_Sam112, members of a new bacteriophage genus with unusual small terminase structure
Source: Sci Rep. 2021 Jun 9;11:12173. doi: 10.1038/s41598-021-91289-x (PMC8190038; doi:10.1038/s41598-021-91289-x)
Supplement: Supplementary file 1 — Supplementary Information 1. [file 41598_2021_91289_MOESM1_ESM.pdf]

vB_BcM_Sam46 and vB_BcM_Sam112, members of a new bacteriophage genus with unusual small terminase structure

Olesya A. Kazantseva, Emma G. Piligrimova and Andrey M. Shadrin

Laboratory of Bacteriophage Biology, Skryabin Institute of Biochemistry and Physiology of Microorganisms, RAS, 142290, Pushchino, Russia; olesyakazantseva@bk.ru (O.A.K.); e.piligrimova@ibpm.ru (E.G.P.); andrey2010s@gmail.com (A.M.S.)

* Correspondence: olesyakazantseva@bk.ru (O.A.K.), andrey2010s@gmail.com (A.M.S.)

Supplementary Information:

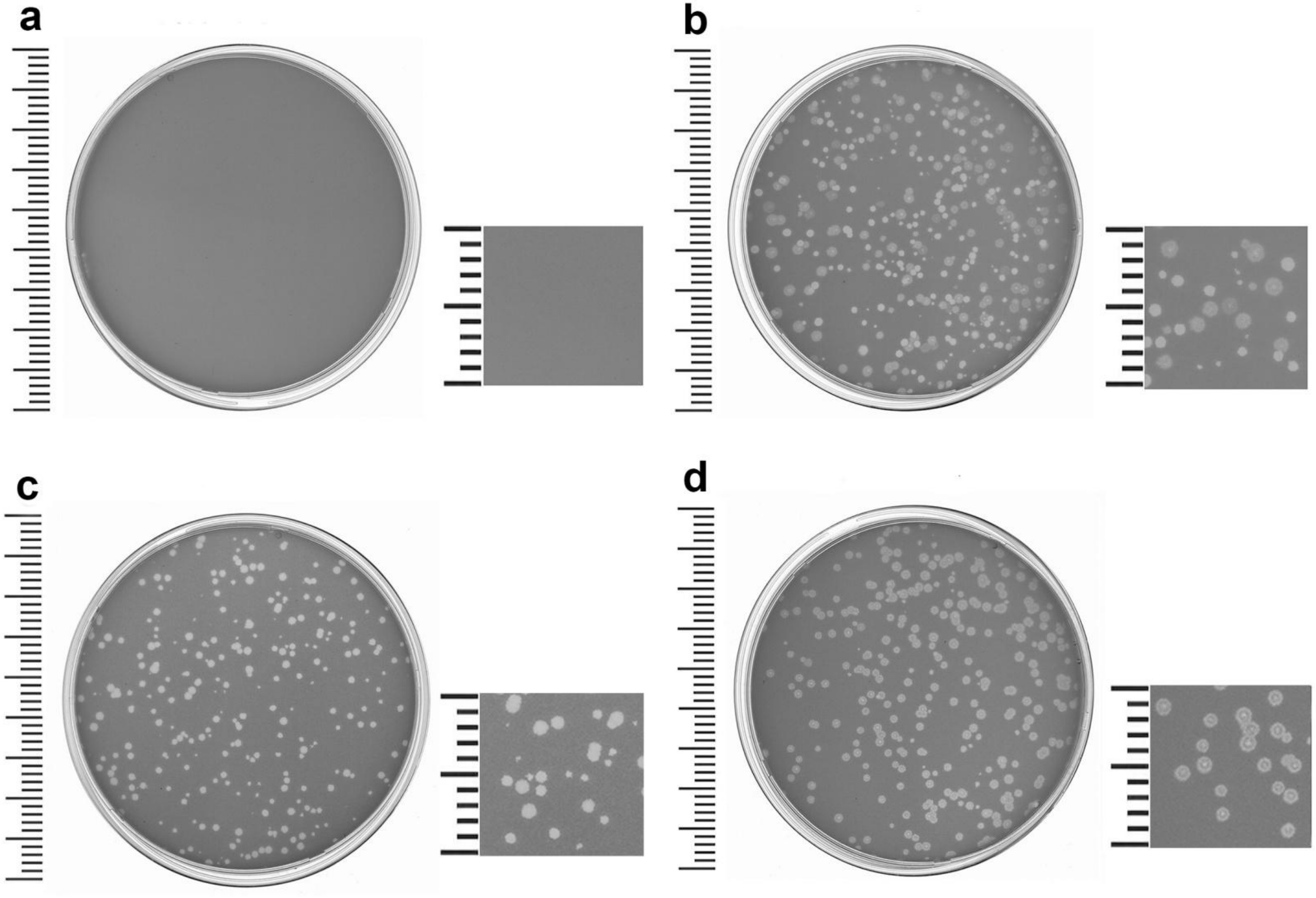

Fig. S1. *Bacillus* phage Sam46 plaques morphology on the lawn of the strain *B. cereus* VKM B-370. **a** – control plate (without phage); **b** – *B. cereus* VKM B-370 infected with original Sam46 phage; **c** – *B. cereus* VKM B-370 infected with purified Sam46-C preparation; **d** – *B. cereus* VKM B-370 infected with purified Sam46-T preparation.

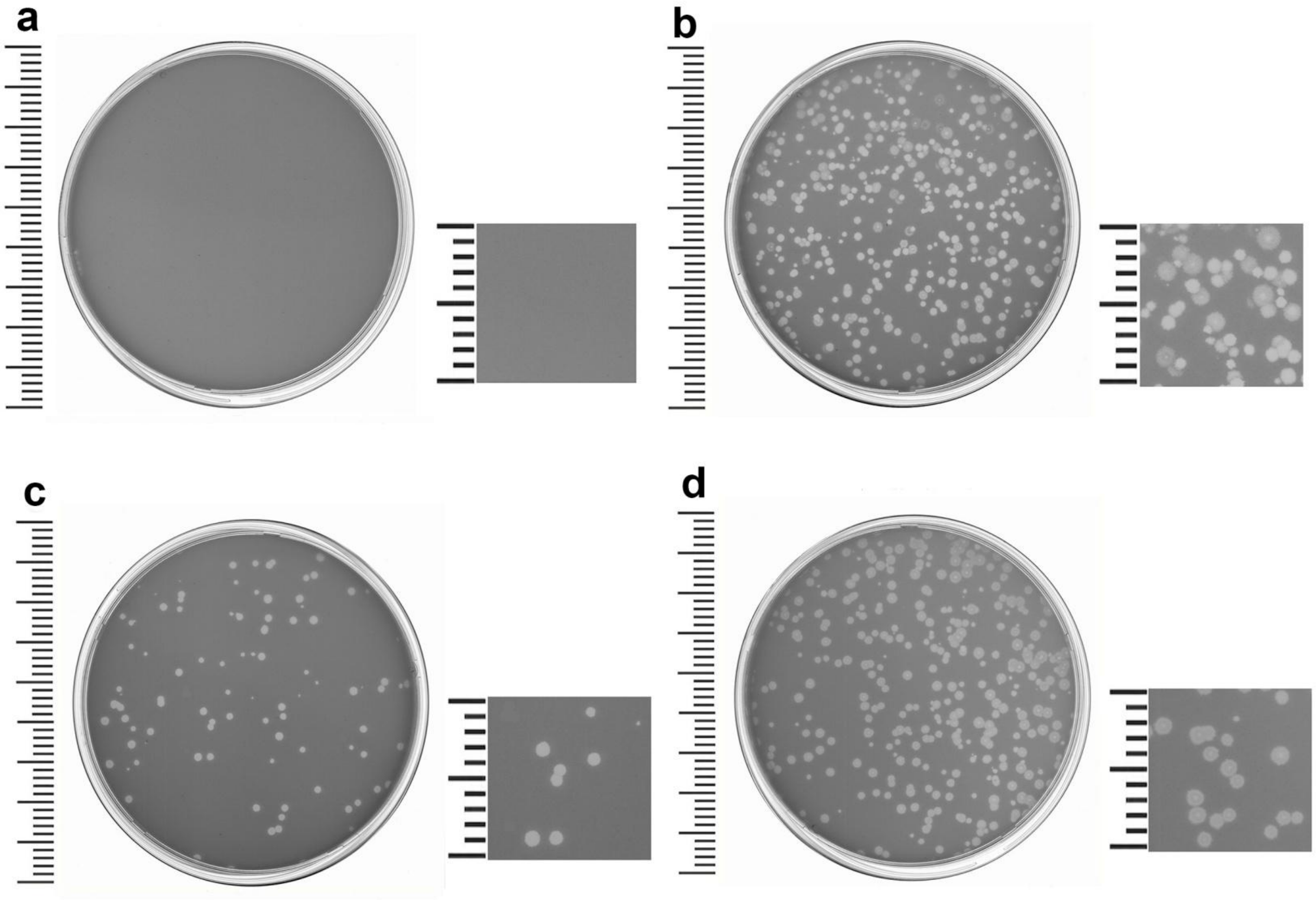

Fig. S2. *Bacillus* phage Sam112 plaques morphology on the lawn of the strain *B. cereus* VKM B-370. **a** – control plate (without phage); **b** – *B. cereus* VKM B-370 infected with original Sam112 phage; **c** – *B. cereus* VKM B-370 infected with purified Sam112-C preparation; **d** – *B. cereus* VKM B-370 infected with purified Sam112-T preparation.

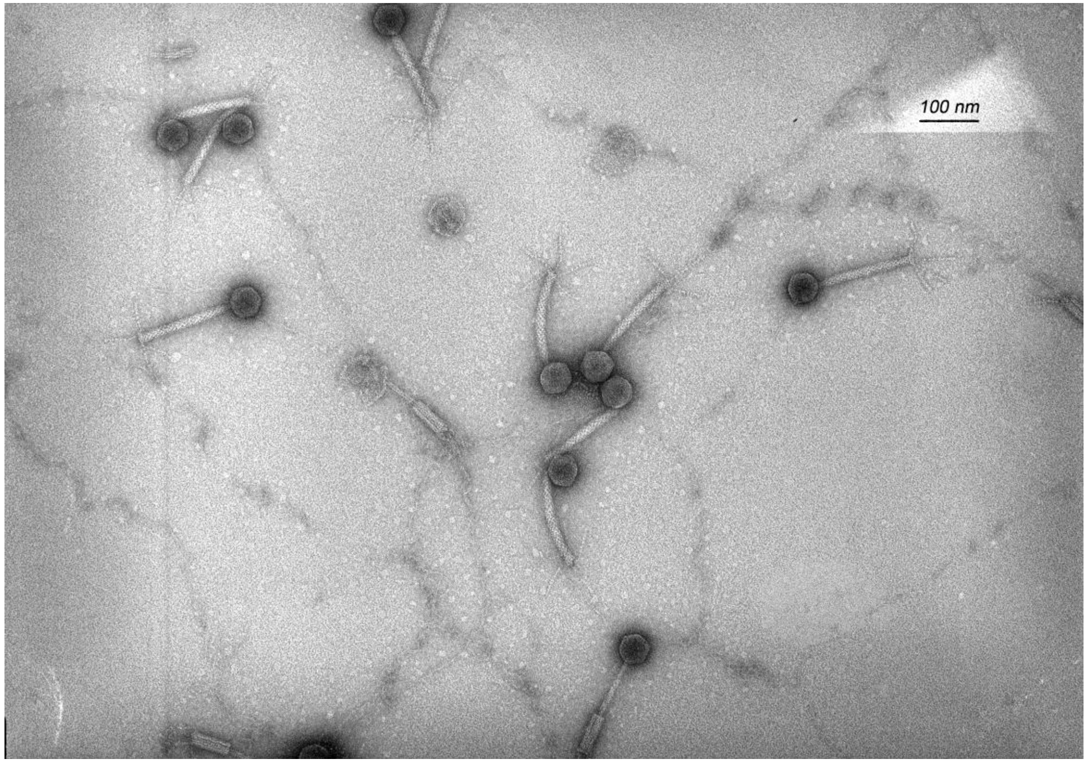

Fig. S3. Transmission electron microscopy of *Bacillus* phage Sam46. This is the original TEM micrograph used to generate Figure 1a in the main text. The image was taken using a JEM-100 (JEOL, Japan) transmission electron microscope at 80 kV accelerating voltage on Kodak film SO-163 (Kodak, Cat. # 74144, Hatfield, PA, USA).

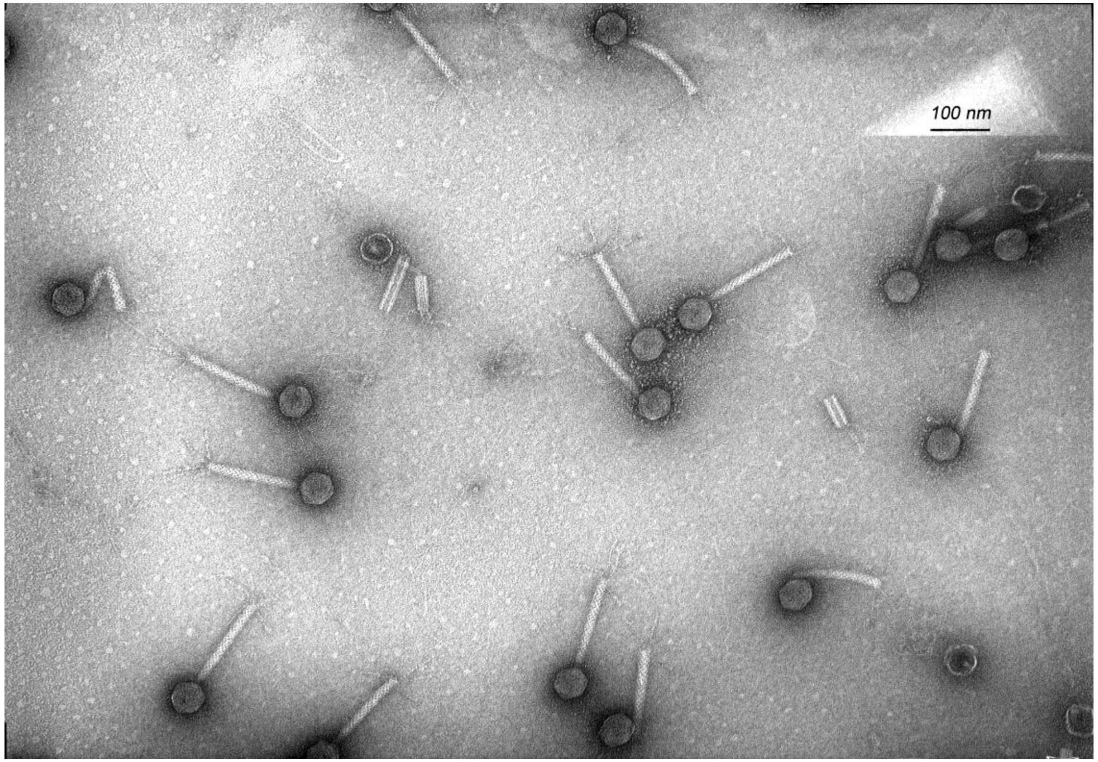

Fig. S4. Transmission electron microscopy of *Bacillus* phage Sam112. This is the original TEM micrograph used to generate Figure 1b in the main text. The image was taken using a JEM-100 (JEOL, Japan) transmission electron microscope at 80 kV accelerating voltage on Kodak film SO-163 (Kodak, Cat. # 74144, Hatfield, PA, USA).

Table S1. Annotation of *Bacillus* phage Sam46

ORF№	Start codon	Stop codon	Strand	Blast results		Conserved domains, Blast		Hhpred results	Annotation
				**Name**	**E-val**	**Name, (region)**	**E-val**	**(Prob./E-val)**	
1	1	765	+	terminase small subunit [*Thermus* phage phi OH2]<br>terminase small subunit [*Bacillus* phage phi4B1]<br>terminase small subunit [*Brevibacillus* phage Davies]	2.0e-43<br>7.0e-39<br>5.0e-37	Ftsk_gamma (11-69)<br>Terminase_2 (107-230)<br>Ftsk_gamma (11-70)	1.24e-28<br>1.29e-27<br>2.25e-27	PF03592.17; ; Terminase_2 ; Terminase small subunit(99.84/5.90e-20)<br>COG3728; XtmA; Phage terminase, small subunit [Mobilome: prophages, transposons].(99.76/1.20e-17)<br>d2j5pa1; a.4.5.67 (A:1261-1329) DNA translocase FtsK {*Escherichia coli* [TaxId: 562]}(99.6/5.30e-15)	FtsK gamma domain-containing small terminase subunit
2	746	2020	+	terminase large subunit [*Brevibacillus* phage Davies]<br>putative terminase large subunit [*Brevibacillus* phage Osiris]<br>terminase large subunit [*Thermus* phage phi OH2]	0.0e+00<br>0.0e+00<br>0.0e+00	XtmB (31-417)<br>Terminase_3 (31-233)	1.99e-61<br>7.89e-41	COG1783; XtmB; Phage terminase large subunit [Mobilome: prophages, transposons].(100.0/5.90e-39)<br>PF03354.16; ; Terminase_1 ; Phage Terminase(100.0/2.70e-33)<br>COG5323; COG5323; Large terminase phage packaging protein [Mobilome: prophages, transposons].(100.0/8.20e-33)	large terminase subunit

3	2036	3478	+	phage portal protein, SPP1 [*Staphylococcus* phage SpaA1]<br>putative portal protein [uncultured *Caudovirales* phage]<br>phage portal protein [*Staphylococcus* phage StauST398-3]	1.0e-112<br>1.0e-100<br>3.0e-87	Phage_prot_Gp6 (48-437)	2.23e-74	PF05133.15; ; Phage_prot_Gp6 ; Phage portal protein, SPP1 Gp6-like(100.0/9.10e-37)<br>PF16510.6; ; P22_portal ; Phage P22-like portal protein(99.56/4.10e-12)<br>PF12236.9; ; Head-tail_con ; Bacteriophage head to tail connecting protein(99.5/3.30e-11)	portal protein
4	3478	4398	+	minor head protein [Deep-sea *thermophilic* phage D6E]<br>minor capsid protein [*Aeribacillus* phage AP45]<br>minor head structural protein [*Exiguobacterium* phage vB_EauM-23]	4.0e-106<br>2.0e-97<br>1.0e-63	Phage_Mu_F (150-273)<br>COG2369 (64-296)<br>phageSPP1_gp7 (151-276)	1.44e-14<br>1.33e-05<br>9.36e-04	PF06152.12; ; Phage_min_cap2 ; Phage minor capsid protein 2(99.92/2.60e-23)<br>COG5585; COG5585; NAD+--asparagine ADP-ribosyltransferase [Signal transduction mechanisms].(99.89/1.30e-21)<br>COG2369; COG2369; Uncharacterized conserved protein, contains phage Mu gpF-like domain [Function unknown].(99.84/4.20e-18)	minor capsid protein
5	4532	5242	+	phage scaffold protein [*Staphylococcus* phage SpaA1]<br>gp8 [*Brochothrix* phage BL3]	4.0e-12<br>2.0e-09	-	-	PF14265.7; ; DUF4355 ; Domain of unknown function (DUF4355)(99.79/3.70e-17)	DUF4355-containing protein, putative scaffold protein

				putative scaffold protein [Oenococcus phage phiS11]	2.0e-04				
6	5259	6248	+	capsid protein [Phage Altai3]<br>coat protein [*Anoxybacillus* phage A403]<br>coat protein [*Enterococcus* phage phiFL3A]	2.0e-156<br>4.0e-143<br>3.0e-99	-	-	PF13252.7; ; DUF4043 ; Protein of unknown function (DUF4043)(99.66/5.00e-15) PF11651.9; ; P22_CoatProtein ; P22 coat protein - gene protein 5(99.37/3.80e-12) d2fsya1; d.183.1.1 (A:104-383) Major capsid protein gp5 {Bacteriophage HK97 [TaxId: 37554]}(99.33/4.90e-11)	major capsid protein
7	6323	6568	+	hp	hp	-	-	PF09124.11; ; Endonuc-dimeris ; T4 recombination endonuclease VII, dimerisation(89.41/1.30e+00)	hp
8	6555	6881	+	putative head morphogenesis protein [uncultured *Caudovirales* phage]<br>head-tail connector protein [*Aeribacillus* phage AP45]	2.0e-08<br>3.0e-07	gp15 (29-101)	1.02e-04	PF05135.14; ; Phage_connect_1 ; Phage gp6-like head-tail connector protein(99.68/9.40e-16) cd08051; gp6_gp15_like; Head-Tail Connector Proteins gp6 and gp15, and similar proteins. Members of this family include the proteins gp6 and gp15 from bacteriophage HK97 and SPP1, respectively.(99.64/5.00e-15) PF11436.9; ; DUF3199 ; Protein of unknown function	gp15-like head completion protein

								(DUF3199)(99.63/2.20e-14)	
9	6881	7507	+	putative morphogenesis protein [*Lactobacillus* phage LBR48]<br>gp11 [*Listeria* phage B054]<br>neck protein [*Vibrio* phage 1.054.O._10N.261.52.A1]	1.0e-34<br>9.0e-18<br>2.0e-11	-	-	PF05069.14; ; Phage_tail_S ; Phage virion morphogenesis family(98.13/9.20e-06) COG5005; COG5005; Mu-like prophage protein gpG [Mobilome: prophages, transposons].(97.26/2.60e-03) PF05069.14; ; Phage_tail_S ; Phage virion morphogenesis family(96.57/2.60e-03)	tail completion protein
10	7507	7857	+	hp	hp	-	-	PF12206.9; ; DUF3599 ; Domain of unknown function (DUF3599)(98.95/3.60e-08) COG5614; COG5614; Bacteriophage head-tail adaptor [Mobilome: prophages, transposons].(98.87/1.30e-07) PF05521.12; ; Phage_H_T_join ; Phage head-tail joining protein(98.46/5.70e-06)	XkdH-like head completion protein
11	7848	8342	+	gp13 [*Listeria* phage B054]	3.0e-04	-	-	PF13554.7; ; DUF4128 ; Bacteriophage related domain of unknown function(96.8/5.40e-02)	hp
12	8358	9365	+	structural protein [*Listeria* phage PSU-VKH-LP041]	8.0e-44	DUF3383<br>(101-333)	5.88e-19	PF11863.9; ; DUF3383 ; Protein of unknown function (DUF3383)(100.0/5.60e-46)	tail sheath protein

				gp14 [*Listeria* phage B054]<br>tail sheath protein [*Acinetobacte*r phage AM24]	9.0e-44<br>1.0e-31			COG4386; COG4386; Mu-like prophage tail sheath protein gpL [Mobilome: prophages, transposons].(99.88/1.40e-20) PF10758.10; ; DUF2586 ; Protein of unknown function (DUF2586)(99.67/3.50e-14)	
13	9378	9785	+	phage structure protein [Deep-sea *thermophilic* phage D6E]<br>structure protein [*Aeribacillus* phage AP45]<br>putative structural protein [*Pseudomonas* phage POR1]	1.0e-45<br>4.0e-17<br>8.0e-09	DUF3277<br>(17-74)	6.23e-07	PF11681.9; ; DUF3277 ; Protein of unknown function (DUF3277)(99.97/1.60e-29) PF07316.12; ; DUF1463 ; Protein of unknown function (DUF1463)(98.65/7.10e-07) d2guja1; b.106.1.3 (A:5-143) Phage-like element PBSX protein XkdM {*Bacillus* subtilis [TaxId: 1423]}(97.21/2.70e-02)<br>PF10618.9 Tail_tube; Phage tail tube protein gpM-like phage Mu (96.11/0.087)	tail tube protein
14	9838	10113	+	-	-	-	-	PF12363.9; ; Phage_TAC_12 ; Phage tail assembly chaperone protein, TAC(89.36/4.00e+00)	putative tail assembly chaperone
15	10145	10330	+	-	-	-	-	PF06528.13; ; Phage_P2_GpE ; Phage P2 GpE(85.69/9.00e-01)	putative tail assembly chaperone

16	10335	13421	+	tape measure protein [*Bacillus* phage 056SW001B]<br>tape measure protein [*Bacillus* phage 019DV002]<br>putative tape measure protein [*Staphylococcus* phage SP5]	4.0e-47<br>4.0e-47<br>8.0e-44	COG5412 (618-905)<br>COG5412 (359-739)	1.70e-12<br>9.46e-07	COG5412; COG5412; Phage-related protein [Mobilome: prophages, transposons].(99.75/1.90e-11) COG5283; COG5283; Phage-related tail protein [Mobilome: prophages, transposons].(99.76/4.00e-09) COG5412; COG5412; Phage-related protein [Mobilome: prophages, transposons].(99.54/6.50e-08) COG5280 YqbO; Phage-related minor tail protein [Mobilome: prophages, transposons]. (99.23/9,7e-06)	putative tail tape measure protein
17	13435	14043	+	LysM domain/BON superfamily protein [*Exiguobacterium* phage vB_EauM-23]<br>muramidase [*Aeribacillus* phage AP45]<br>XkdP protein [*Clostridium* virus phiCD119]	3.0e-25<br>8.0e-14<br>2.0e-08	PRK11198 (145-202)<br>LysM (150-201)	2.50e-19<br>1.12e-8	COG3499; COG3499; Phage protein U [Mobilome: prophages, transposons].(99.23/1.10e-10) PF06995.12; ; Phage_P2_GpU ; Phage P2 GpU(98.71/2.40e-07) COG1652; XkdP; Nucleoid-associated protein YgaU, contains BON and LysM domains [Function unknown].(98.57/3.60e-07)	LysM domain-containing peptidoglycan-binding protein
18	14056	14373	+	hp	hp	-	-	PF10657.10; ; RC-P840_PscD ; Photosystem P840 reaction cœntre protein PscD(71.24/6.70e+00)	hp
19	14366	15346	+	gp21 [*Listeria* phage B054]<br>putative baseplate hub	1.0e-35	-	-	COG4379; COG4379; Mu-like prophage tail protein gpP	gpP-like baseplate hub protein

				[Enterobacteria phage ECGD1]<br>Phi92_gp137 [Enterobacteria phage phi92]	3.0e-10<br>3.0e-10			[Mobilome: prophages, transposons].(99.9/3.70e-21) COG3500; gpD; Phage protein D [Mobilome: prophages, transposons].(99.89/1.50e-20) COG3501; VgrG; Uncharacterized conserved protein, implicated in type VI secretion and phage assembly [Intracellular trafficking, secretion, and vesicular transport, Mobilome: prophages, transposons, General function prediction only].(99.85/3.50e-19)	
20	15359	15838	+	baseplate central spike protein [*Acinetobacte*r phage BS46]<br>putative baseplate protein [*Acinetobacte*r phage YMC13/03/R2096]<br>putative spike protein [Prokaryotic dsDNA virus sp.]	3.0e-07<br>7.0e-07<br>3.0e-06	Gp138_N<br>(25-108)	1.08e-16	PF18352.2; ; Gp138_N ; Phage protein Gp138 N-terminal domain(99.82/2.60e-19) COG4540; gpV; Phage P2 baseplate assembly protein gpV [Mobilome: prophages, transposons].(99.63/5.60e-14) COG4384; gp45; Mu-like prophage protein gp45 [Mobilome: prophages, transposons].(99.56/4.50e-13)	gpV-like tail spike (tail needle)
21	15838	16206	+	putative oxidoreductase [*Burkholderia* phage Bups phi1]<br>putative baseplate	6.0e-06	DUF2634<br>(2-104)	9.25e-05	PF10934.9; ; DUF2634 ; Protein of unknown function (DUF2634)(99.82/8.50e-19) COG4381; gp46; Mu-like prophage	gp25-like baseplate protein

				component [Pantoea phage vB_PagM_AAM37]<br>DUF2634 domain-containing protein [*Listeria* phage PSU-VKH-LP041]	3.0e-04<br>6.0e-04	GPW_gp25 superfamily<br>(2-104)	2.75e-5	protein gp46 [Mobilome: prophages, transposons].(99.71/9.80e-16) COG3628; COG3628; Phage baseplate assembly protein W [Mobilome: prophages, transposons].(99.61/8.70e-14) PF04965.15; GPW_gp25 ; Gene 25-like lysozyme (99.58/1.2e-13)	
22	16193	17374	+	baseplate protein [*Aeribacillus* phage AP45]<br>baseplate protein [Deep-sea *thermophilic* phage D6E]<br>putative baseplate protein [uncultured *Caudovirales* phage]	1.0e-71<br>2.0e-69<br>5.0e-61	Baseplate_J<br>(74-188)<br>JayE<br>(6-391)<br>TIGR02243<br>(144-355)	5.98e-23<br>6.40e-22<br>1.99e-05	COG3299; JayE; Uncharacterized phage protein gp47/JayE [Mobilome: prophages, transposons].(100.0/9.00e-37) COG3948; COG3948; Phage-related baseplate assembly protein [Mobilome: prophages, transposons].(99.97/5.50e-32) PF04865.15; ; Baseplate_J ; Baseplate J-like protein(99.97/2.20e-29)	J-like baseplate protein
23	17371	17997	+	gp25 [*Listeria* phage B054]<br>putative baseplate protein [*Lactobacillus* phage LBR48]<br>baseplate protein [*Lactobacillus* phage 3-	1.0e-32<br>1.0e-14<br>3.0e-06	-	-	PF11041.9; ; DUF2612 ; Protein of unknown function (DUF2612)(99.76/5.30e-18) PF10076.10; ; DUF2313 ; Uncharacterised protein conserved in bacteria (DUF2313)(99.69/3.10e-15) COG3778; YmfQ; Uncharacterized	I-like baseplate protein

				SAC12]				protein YmfQ in lambdoid prophage, DUF2313 family [Mobilome: prophages, transposons].(99.55/4.40e-13) COG4385 gpI; Bacteriophage P2-related tail formation protein [Mobilome: prophages, transposons]. (99.35/1.6e-11)	
24	18013	21414	+	tail fiber protein [Deep-sea *thermophilic* phage D6E]<br>tail fiber protein [*Aeribacillus* phage AP45]<br>YomR-like protein [*Bacillus* phage Shbh1]	2.0e-19<br>4.0e-17<br>8.0e-12	Phage_fiber_2 (328-365)<br>C1Q (1028-1117)	4.86e-08<br>3.60e-04	cd16446; TNFSF_C1q; complement component 1, q subcomponent-like (C1ql), a component of C1q/TNF superfamily.(98.18/2.90e-05) cd16447; TNFSF_C1q-like; C1q-like family containing adiponectin, and C-terminal NCI domains of collagen VIII and X.(97.68/6.40e-04) PF00386.22; ; C1q ; C1q domain(96.83/3.80e-02)	gpH-like tail fiber receptor binding protein
25	21430	21714	+	hp	hp	XkdW (4-75)	4.79e-08	PF09636.11; XkdW ; XkdW protein(99.79/2.90e-18) d2hg7a1; d.186.2.1 (A:1-60) Phage-like element PbsX protein XkdW {*Bacillus subtilis* [TaxId: 1423]}(99.73/3.00e-17)	XkdW-like protein
26	21730	21963	+	-	-	-	-	PF09693.11; Phage_XkdX ; Phage uncharacterised protein (Phage_XkdX)(97.19/1.30e-03) gp23.1 (SPP1) putative chaperone	putative chaperone

								(97.6/e-6)	
27	22080	22379	+	XpaF1 protein [*Bacillus* phage vB_BtS_BMBtp3]<br>XhlA hemolysin [*Clostridium* phage phiCT19406C]<br>hemolysin XhlA protein [*Bacillus* phage vB_BtS_BMBtp14]	2.0e-03<br>3.0e-03<br>3.0e-03	XhlA<br>(21-90)	1.67e-09	PF10779.10; ; XhlA ; Haemolysin XhlA(99.31/8.40e-11)<br>PF07439.12; ; DUF1515 ; Protein of unknown function (DUF1515)(98.4/6.40e-05)<br>PF11166.9; ; DUF2951 ; Protein of unknown function (DUF2951)(97.78/3.10e-03)	XhlA domain-containing holin-like protein
28	22382	23188	+	endolysin [Sporosarcina phage Lietuvens]<br>gp22 [*Bacillus* virus G]<br>PlyM14 [uncultured phage]	1.0e-71<br>7.0e-70<br>1.0e-66	MurNAc-LAA<br>(2-181)<br>Ami_3<br>(67-180)	2.50e-40<br>1.06e-20	d1jwqa_; c.56.5.6 (A:) N-acetylmuramoyl-L-alanine amidase CwlV {*Paenibacillus* polymyxa [TaxId: 1406]}(99.97/7.10e-28)<br>COG0860; AmiC; N-acetylmuramoyl-L-alanine amidase [Cell wall/membrane/envelope biogenesis].(99.96/6.50e-27)<br>d1xova2; c.56.5.6 (A:1-180) Endolysin Ply, catalytic domain {Bacteriophage Psa [TaxId: 171618]}(99.94/1.00e-24)	N-acetylmuramoyl-L-alanine amidase
29	23200	23421	+	SPP1 family holin [*Bacillus* phage Waukesha92]<br>lysis protein [*Bacillus* phage vB_BthS-TP21T]	4.0e-25<br>2.0e-15	holin_SPP1<br>(1-72)	6.13e-12	PF04688.14; ; Holin_SPP1 ; SPP1 phage holin(99.91/1.00e-23)<br>COG5546; COG5546; Uncharacterized membrane protein [Function unknown].(96.8/3.20e-02)	holin

				putative holin [*Bacillus* phage PBC4]	6.0e-15				
30	23570	24283	+	hp	hp	-	-	('-', '-')	hp
31	24297	24476	+	-	-	-	-	d1rh6a_ a.6.1.7 (A:) Excisionase Xis {Bacteriophage lambda [TaxId: 10710]} (98.74/9.8e-8) PF11112.9; ; PyocinActivator ; Pyocin activator protein PrtN(98.87/2.60e-08) PF10743.10; ; Phage_Cox ; Regulatory phage protein cox(98.85/2.70e-08) PF06806.13; DUF1233 ; Putative excisionase (DUF1233) (98.71/3.6e-7)	DNA-binding protein
32	24466	24660	+	hp	hp	-	-	('-', '-')	hp
33	24657	24764	+	hp	hp	-	-	PF15168.7; ; TRIQK ; Triple QxxK/R motif-containing protein family(81.06/2.00e+00)	hp
34	24805	25206	+	putative membrane lipoprotein [*Paenibacillus* phage Yerffej]	7.0e-04	-	-	COG2913; BamE; Outer membrane protein assembly factor BamE, lipoprotein component of the BamABCDE complex [Cell wall/membrane/envelope biogenesis].(99.24/3.30e-10)	hp

								PF12978.8; ; DUF3862 ; Domain of Unknown Function with PDB structure (DUF3862)(99.14/1.00e-09)<br>PF07467.12; ; BLIP ; Beta-lactamase inhibitor (BLIP)(98.9/2.30e-08)	
35	25350	25838	+	homing endonuclease [Podoviridae sp. ctviO18]<br>HTH homing endonuclease [*Staphylococcus* phage Stau2]<br>deoxyuridine 5'-triphosphate nucleotidohydrolase family protein [*Bacillus* phage Bastille]	7.0e-21<br>9.0e-20<br>1.0e-18	-	-	d1u3em2; d.285.1.1 (M:106-174) Intron-encoded homing endonuclease I-HmuI {Bacteriophage SPO1 [TaxId: 10685]}(98.87/4.30e-08)<br>d1u3em1; d.4.1.3 (M:1-105) Intron-encoded homing endonuclease I-HmuI {Bacteriophage SPO1 [TaxId: 10685]}(98.19/2.90e-06)<br>PF13392.7; ; HNH_3 ; HNH endonuclease(97.88/2.20e-05)	HNH endonuclease
36	25844	26554	+	hp	hp	-	-	('-', '-')	hp
37	26557	26703	+	hp	hp	-	-	PF09538.11; ; FYDLN_acid ; Protein of unknown function (FYDLN_acid)(96.99/3.70e-04)<br>COG1675; TFA1; Transcription initiation factor IIE, alpha subunit [Transcription].(96.83/9.90e-04)<br>PF11023.9; ; DUF2614 ; Zinc-ribbon containing domain(95.17/8.10e-03)	hp

38	26707	26850	+	hp	hp	-	-	('-', '-')	hp
39	26863	27063	+	-	-	-	-	COG1254; AcyP; Acylphosphatase [Energy production and conversion].(97.0/8.20e-03) d1apsa_; d.58.10.1 (A:) Acylphosphatase {Horse (*Equus caballus*) [TaxId: 9796]}(96.92/1.20e-02) d1ulra_; d.58.10.1 (A:) Acylphosphatase {*Thermus thermophilus* [TaxId: 274]}(96.81/1.50e-02)	acylphosphatase
40	27068	27271	+	hp	hp	-	-	PF14122.7; ; YokU ; YokU-like protein, putative antitoxin(77.07/1.40e+00)	hp
41	27413	27700	+	glutaredoxin-like protein [*Bacillus* phage Wes44]<br>glutaredoxin-like protein [*Bacillus* phage Carmen17]	9.0e-44<br>3.0e-42	-	-	d1x93a1; a.43.1.3 (A:31-73) Uncharacterized protein HP0222 {*Helicobacter pylori* [TaxId: 210]}(94.87/1.00e-01) COG4453 COG4453; Uncharacterized conserved protein, DUF1778 family [Function unknown].(92.97/0.41) d1baza_ a.43.1.1 (A:) Arc repressor {*Salmonella* bacteriophage P22 [TaxId: 10754]} (88.38/2.4) d2cpga_ a.43.1.3 (A:) Transcriptional repressor CopG	CopG/Arc/MetJ DNA-binding domain-containing protein

								{*Streptococcus agalactiae* [TaxId: 1311]} (87.44/2.7)	
42	27693	28046	+	putative glycosyltransferase [*Bacillus* phage vB_BspS_SplendidRed]	5.0e-36	-	-	PF04956.14; ; TrbC ; TrbC/VIRB2 pilin(97.42/2.20e-03) PF18895.1; ; T4SS_pilin ; Type IV secretion system pilin(96.38/6.60e-02)	hp
43	28061	28414	+	hp	hp	-	-	('-', '-')	hp
44	28411	30624	+	ATPase domain protein [*Bacillus* phage 049ML001]<br><br>ATPase domain protein [*Bacillus* phage 049ML003]<br><br>ATPase domain protein [*Bacillus* phage 000TH010]	0.0e+00<br><br>0.0e+00<br><br>0.0e+00	-	-	d1e9ra_; c.37.1.11 (A:) Bacterial conjugative coupling protein TrwB {*Escherichia coli* [TaxId: 562]}(99.94/8.60e-25) PF19044.1; ; P-loop_TraG ; TraG P-loop domain(99.94/2.50e-24) PF05872.13; DUF853 ; Bacterial protein of unknown function (DUF853)(99.92/1.90e-23)<br><br>PF12846.8; AAA_10 ; AAA-like domain (99.93/2.7e-23)	ATPase domain containing protein
45	30950	31090	+	hp	hp	-	-	('-', '-')	hp
46	31265	31405	+	-	-	-	-	d1xrxa1; a.43.1.7 (A:1-35) SeqA {*Escherichia coli* [TaxId: 562]}(94.91/5.10e-02) PF17206.4; SeqA_N ; SeqA protein N-terminal domain(94.45/9.30e-02) PF10723.10; RepB-RCR_reg ;	putative transcriptional repressor

								Replication regulatory protein RepB (89.55/1.2)	
47	31424	32785	+	exonuclease [*Bacillus* phage Ray17]<br>5'-3' exonuclease [*Bacillus* phage 000TH010]<br>5'-3' exonuclease [*Bacillus* phage 049ML003]	2.0e-87<br>6.0e-87<br>2.0e-85	COG5377 (11-322)<br>YqaJ (24-171)<br>PRK00409 (313-415)	6.79e-62<br>3.31e-35<br>2.64e-04	COG5377; COG5377; Phage-related protein, predicted endonuclease [Mobilome: prophages, transposons].(100.0/3.50e-40) d3sm4a_; c.52.1.13 (A:) lambda exonuclease {Bacteriophage lambda [TaxId: 10710]}(99.95/7.30e-27) PF09588.11; YqaJ ; YqaJ-like viral recombinase domain(99.74/1.90e-17)	YqaJ-domain containing exonuclease
48	32787	33023	+	hp	hp	-	-	PF18077.2; ; DUF5595 ; Domain of unknown function (DUF5595)(95.37/2.10e-01)	hp
49	33025	33870	+	RecT [*Geobacillus* phage GBK2]<br>recombinase [*Bacillus* phage 049ML001]<br>putative recombinase [*Bacillus* phage vB_BspS_SplendidRed]	6.0e-112<br>6.0e-105<br>2.0e-103	recT (23-279)<br>rect (2-250)<br>RecT (56-253)	2.18e-114<br>2.54e-83<br>2.83e-51	COG3723; RecT; Recombinational DNA repair protein RecT [Replication, recombination and repair].(100.0/5.80e-42) PF03837.15; RecT ; RecT family(100.0/1.10e-30)	recombination protein RecT

50	33883	34341	+	putative single-stranded DNA-binding protein [*Listeria* phage PSU-VKH-LP019]<br>putative SSB protein [*Listeria* phage A118]<br>gp45 [*Brochothrix* phage NF5]	4.0e-69<br>1.0e-66<br>3.0e-65	PRK06751 (1-152)<br>SSB (2-102)<br>Ssb (1-152)	3.06e-71<br>3.33e-49<br>1.73e-47	COG0629; Ssb; Single-stranded DNA-binding protein [Replication, recombination and repair].(99.92/2.40e-22)<br>d1qvca_; b.40.4.3 (A:) ssDNA-binding protein {*Escherichia coli* [TaxId: 562]}(99.91/1.90e-21)<br>KOG1653; Single-stranded DNA-binding protein [Replication, recombination and repair](99.83/1.80e-18)	SSBP (single-stranded DNA binding protein)
51	34354	34488	+	hp	hp	-	-	PF07874.12; ; DUF1660 ; Prophage protein (DUF1660)(98.49/6.00e-08)	hp
52	34501	34665	+	hp	hp	-	-	PF10058.10; ; zinc_ribbon_10 ; Predicted integral membrane zinc-ribbon metal-binding protein(70.42/4.50e+00)	hp
53	34716	35369	+	DNA polymerase III subunit beta [*Bacillus* phage Silence]<br>DNA polymerase III subunit beta [*Psychrobacillus* phage Perkons]<br>DNA polymerase III subunit beta	4.0e-60<br>3.0e-08<br>4.0e-07	beta_clamp (26-215)<br>DNA_pol3_beta_3 (126-214)<br>DnaN	3.11e-10<br>4.19e-05<br>8.93e-05	cd00140; beta_clamp; Beta clamp domain. The beta subunit (processivity factor) of DNA polymerase III holoenzyme, refered to as the beta clamp, forms a ring shaped dimer that encircles dsDNA (sliding clamp) in bacteria.(99.94/1.70e-24)<br>COG0592; DnaN; DNA polymerase III sliding clamp (beta) subunit,	PolIIIβ-like processivity factor (DNA clamp)

				[*Staphylococcus* phage SPbeta-like]		(79-216)		PCNA homolog [Replication, recombination and repair].(99.85/2.30e-19) d1vpka3; d.131.1.1 (A:244-366) DNA polymerase III, beta subunit {*Thermotoga maritima* [TaxId: 2336]}(99.73/2.40e-16)	
54	35397	35924	+	hp	hp	-	-	('-', '-')	hp
55	35924	36250	+	hp	hp	-	-	('-', '-')	hp
56	36252	36536	+	phage related protein [*Staphylococcus* phage SpaA1]	7.0e-14	-	-	PF04009.13; ; DUF356 ; Protein of unknown function (DUF356)(81.87/6.10e+00)	hp
57	36548	36868	+	hp	hp	-	-	COG3462; COG3462; Uncharacterized membrane protein [Function unknown].(78.61/2.70e+00)	hp
58	36869	37042	+	hp	hp	-	-	PF12208.9; ; DUF3601 ; Domain of unknown function (DUF3601)(87.76/1.10e+00)	hp
59	37175	37348	+	DNA-binding protein [*Bacillus* phage Silence]<br>DNA binding domain protein [*Bacillus* phage 049ML001]	9.0e-17<br>3.0e-11	HTH_17<br>(4-52)	1.81e-09	d1j9ia_; a.6.1.5 (A:) Terminase gpNU1 subunit domain {Bacteriophage lambda [TaxId: 10710]}(99.05/1.50e-08) PF11112.9; ; PyocinActivator ; Pyocin activator protein PrtN(99.02/2.10e-08)	DNA-binding protein

				DNA-binding protein [*Lysinibacillus* phage vB_LspM-01]	1.0e-10			PF10743.10; ; Phage_Cox ; Regulatory phage protein cox(99.0/2.50e-08)<br>PF06806.13; DUF1233 ; Putative excisionase (DUF1233) (98.72/4.7e-7)<br>COG4220 Nu1; Phage DNA packaging protein, Nu1 subunit of terminase [Mobilome: prophages, transposons]. (98.72/6.1e-7)	
60	37348	37551	+	hp	hp	-	-	('-', '-')	hp
61	37560	38354	+	DNA replication protein [*Anoxybacillus* phage A403]<br>replication initiation protein [*Staphylococcus* phage phi879]<br>replication initiation protein [*Staphylococcus* phage phi575]	6.0e-47<br>1.0e-40<br>1.0e-39	-	-	PF09681.11; ; Phage_rep_org_N ; N-terminal phage replisome organiser (Phage_rep_org_N)(98.1/1.20e-04)<br>COG2188; MngR; DNA-binding transcriptional regulator, GntR family [Transcription].(97.37/7.60e-04)<br>PF06970.12; ; RepA_N ; Replication initiator protein A (RepA) N-terminus(97.42/8.00e-04)	DnaD domain-containing protein, putative replication initiator
62	38365	38757	+	helicase [*Bacillus* phage Ray17]<br>helicase loader [*Bacillus* phage 000TH010]<br>helicase loader [*Bacillus*	1.0e-10<br>2.0e-10<br>2.0e-10	Inhibitor_G39P<br>(2-63)	1.82e-04	d1no1a1; a.179.1.1 (A:2-67) Replisome organizer (g39p helicase loader/inhibitor protein) {Bacteriophage Spp1 [TaxId: 10724]}(99.58/1.10e-14)<br>PF11417.9; ; Inhibitor_G39P ;	helicase loader

				phage 049ML001]				Loader and inhibitor of phage G40P(99.43/6.60e-13) PF06992.12; ; Phage_lambda_P ; Replication protein P(98.91/9.70e-09)	
63	38754	40082	+	DNA helicase [*Bacillus* phage Silence]<br>helicase [*Geobacillus* phage GBK2]<br>replicative DNA helicase [*Bacillus* phage 11143]	3.0e-131<br>2.0e-98<br>4.0e-98	DnaB (7-435)<br>DnaB_C (175-433)<br>DnaB (7-435)	2.73e-139<br>4.61e-115<br>1.33e-111	COG0305; DnaB; Replicative DNA helicase [Replication, recombination and repair].(100.0/4.60e-39) d1cr1a_; c.37.1.11 (A:) Gene 4 protein (g4p, DNA primase), helicase domain {Bacteriophage T7 [TaxId: 10760]}(99.95/7.30e-25) PF03796.16; ; DnaB_C ; DnaB-like helicase C terminal domain(99.95/1.20e-24)	DnaB-type replicative helicase
64	40075	40413	+	HNH endonuclease I [*Bacillus* phage Slash]<br>DNA binding protein [*Bacillus* phage vB_BthS_BMBphi]<br>HNH homing endonuclease [*Bacillus* phage AvesoBmore]	2.0e-23<br>1.0e-19<br>5.0e-19	HNH_3 (44-88)	1.66e-13	PF05551.12; ; zf-His_Me_endon ; Zinc-binding loop region of homing endonuclease(99.72/5.90e-17) d1u3em1; d.4.1.3 (M:1-105) Intron-encoded homing endonuclease I-HmuI {Bacteriophage SPO1 [TaxId: 10685]}(99.6/5.00e-14) PF13392.7; ; HNH_3 ; HNH endonuclease(99.38/7.50e-13)	HNH endonuclease
65	40531	40821	+	hp	hp	-	-	PF08858.11; ; IDEAL ; IDEAL domain(98.66/5.50e-08)	hp

								COG5582; YpiB; Uncharacterized protein YpiB, UPF0302 family [Function unknown].(96.87/2.60e-03)	
66	40897	42006	+	hp	hp	rfaE_dom_I (35-109)	5.93e-04	KOG1319; bHLHZip transcription factor BIGMAX [Transcription](94.47/1.90e+00)	hp
67	42059	42355	+	-	-	-	-	COG3877; COG3877; Uncharacterized protein, DUF2089 family [Function unknown].(98.89/7.60e-08) PF07750.12; ; GcrA ; GcrA cell cycle regulator(98.66/1.20e-07) d1ijwc_; a.4.1.2 (C:) HIN recombinase (DNA-binding domain) {Synthetic}(98.05/3.50e-05) Phage_antitermQ (97.46/e-5)	putative antitermination protein
68	42361	42588	+	transcription factor [*Bacillus* phage Ray17]	3.0e-03	-	-	PF12677.8; ; DUF3797 ; Domain of unknown function (DUF3797)(95.22/2.60e-02) d1twfi1; g.41.3.1 (I:1-49) RBP9 subunit of RNA polymerase II {Baker's yeast (Saccharomyces cerevisiae) [TaxId: 4932]}(95.43/2.80e-02) cd00629; RNA_pol_M_RPB9_N; RNA_pol_M_RPB9_N. RPB9 is a subunit of eukaryotic RNA	hp

								polymerase II that contributes to transcription elongation by recruiting transcription factor TFIIE to the RNA polymerase II complex.(94.84/6.00e-02)	
69	42585	42851	+	glutaredoxin [*Bacillus* phage Claudi]<br>glutaredoxin [*Bacillus* phage DK2]<br>glutaredoxin [*Bacillus* phage DK3]	6.0e-12<br>3.0e-11<br>1.0e-10	NrdH<br>(5-86)<br>PRK10329<br>(5-54)	1.72e-10<br>1.41e-04	d1zmaa1; c.47.1.1 (A:1-115) Bacterocin transport accessory protein Bta {*Pneumococcus* (*Streptococcus pneumoniae*) [TaxId: 1313]}(99.66/3.90e-15) cd03004; PDI_a_ERdj5_C; PDIa family, C-terminal ERdj5 subfamily; ERdj5, also known as JPDI and macrothioredoxin, is a protein containing an N-terminal DnaJ domain and four redox active TRX domains.(99.68/4.10e-15) d3diea1; c.47.1.1 (A:1-104) Thioredoxin {*Staphylococcus aureus* [TaxId: 1280]}(99.67/5.00e-15)	glutaredoxin-like protein
70	42848	42976	+	hp	hp	-	-	COG0690; SecE; Preprotein translocase subunit SecE [Intracellular trafficking, secretion, and vesicular transport].(90.78/2.20e+00)	hp
71	42990	43124	+	hp	hp	-	-	PF16777.6; ; RHH_7 ; Transcriptional regulator, RHH-like,	hp

								CopG(91.64/6.70e-01)	
72	43127	43642	+	putative dUTPase [uncultured *Caudovirales* phage]<br>dUTP diphosphatase [*Aeribacillus* phage AP45]<br>dUTPase [*Bacillus* phage Silence]	2.0e-36<br>1.0e-34<br>4.0e-32	dUTPase_2 (2-171)<br>NTP-PPase_dUTPase(6-77)<br>56 (138-171)	2.96e-32<br>1.63e-13<br>9.19e-05	COG4508; Dut2; Dimeric dUTPase, all-alpha-NTP-PPase (MazG) superfamily [Nucleotide transport and metabolism].(99.97/2.80e-29) PF08761.12; ; dUTPase_2 ; dUTPase(99.97/3.80e-29) d1w2ya_; a.204.1.1 (A:) Type II deoxyuridine triphosphatase {*Campylobacter jejuni* [TaxId: 197]}(99.95/5.80e-27)	dimeric dUTPase
73	43701	43871	+	hp	hp	-	-	PF04161.14; ; Arv1 ; Arv1-like family(91.83/5.90e-02)	hp
74	43874	44110	+	hp	hp	-	-	PF13397.7; ; RbpA ; RNA polymerase-binding protein(93.09/7.70e-02)	putative sigma-factor
75	44276	44734	+	gp58 [*Listeria* phage A500]<br>holliday junction resolvase [*Bacillus* phage 276BB001]<br>holliday junction resolvase [*Bacillus* phage 019DV002]	1.0e-26<br>2.0e-23<br>6.0e-23	DUF1064 (3-111)	1.62e-35	PF06356.12; DUF1064 ; Protein of unknown function (DUF1064)(99.89/2.30e-21) d1m0da_; c.52.1.17 (A:) Endonuclease I (Holliday junction resolvase) {Bacteriophage T7 [TaxId: 10760]}(99.2/4.90e-10) PF05367.12; ; Phage_endo_I ; Phage endonuclease I(99.11/2.80e-09)	DUF1064 domain-containing protein, putative endonuclease

76	44831	45076	+	hp	hp	-	-	cd17794; TetR_C; Tetr-Family Transcriptional Regulator. TetR; This family of bacterial transcriptional repressors is characterized by the short approximately 50 amino acid stretch of residues constituting the helix-turn-helix DNA binding motif, around the YRFhY motif.(88.12/4.20e+00)	hp
77	45073	45246	+	hp	hp	-	-	cd14788; GumN; poorly characterized family of proteins related to gumN pathogenicity factor of *Xanthomonas*.(67.4/3.70e+00)	hp

Table S2. Annotation of *Bacillus* phage Sam112

ORF№	Start codon	Stop codon	Strand	Blast results		Conserved domains, Blast		Hhpred results	Annotation
				Name	E-val	Name, (region)	E-val	(Prob./E-val)	
1	1	765	+	terminase small subunit [*Thermus* phage phi OH2]<br>terminase small subunit [*Bacillus* phage phi4B1]<br>terminase small subunit [*Brevibacillus* phage Davies]	2.0e-43<br>7.0e-39<br>4.0e-37	Ftsk_gamma (10-69)<br>Terminase_2 (107-230)<br>Ftsk_gamma (11-70)	5.65e-29<br>1.27e-27<br>1.63e-27	PF03592.17; ; Terminase_2 ; Terminase small subunit(99.84/3.80e-20)<br>COG3728; XtmA; Phage terminase, small subunit [Mobilome: prophages, transposons].(99.77/7.50e-18)<br>d2j5pa1; a.4.5.67 (A:1261-1329) DNA translocase FtsK {*Escherichia coli* [TaxId: 562]}(99.64/1.10e-15)	FtsK gamma domain-containing small terminase subunit
2	746	2020	+	terminase large subunit [*Brevibacillus* phage Davies]<br>putative terminase large subunit [*Brevibacillus* phage Osiris]<br>terminase large subunit [*Thermus* phage phi OH2]	0.0e+00<br>0.0e+00<br>0.0e+00	XtmB (31-417)<br>Terminase_3 (31-233)	9.90e-60<br>7.07e-40	COG1783; XtmB; Phage terminase large subunit [Mobilome: prophages, transposons].(100.0/2.30e-39)<br>PF03354.16; ; Terminase_1 ; Phage Terminase(100.0/2.10e-33)<br>COG5323; COG5323; Large terminase phage packaging protein	large terminase subunit

								[Mobilome: prophages, transposons].(100.0/2.30e-33)	
3	2036	3481	+	phage portal protein, SPP1 [*Staphylococcus* phage SpaA1]<br>putative portal protein [uncultured *Caudovirales* phage]<br>phage portal protein [*Staphylococcus* phage StauST398-3]	8.0e-113<br>8.0e-101<br>1.0e-87	Phage_prot_Gp6 (48-437)	1.87e-74	PF05133.15; ; Phage_prot_Gp6 ; Phage portal protein, SPP1 Gp6-like(100.0/2.30e-36)<br>PF16510.6; ; P22_portal ; Phage P22-like portal protein(99.59/1.50e-12)<br>PF12236.9; ; Head-tail_con ; Bacteriophage head to tail connecting protein(99.55/5.60e-12)	portal protein
4	3481	4401	+	minor head protein [Deep-sea *thermophilic* phage D6E]<br>minor capsid protein [*Aeribacillus* phage AP45]<br>minor head structural protein [*Exiguobacterium* phage vB_EauM-23]	3.0e-106<br>3.0e-96<br>1.0e-63	Phage_Mu_F (150-273)<br>COG2369 (64-296)<br>phageSPP1_gp7 (151-276)	1.35e-14<br>2.14e-05<br>8.58e-04	PF06152.12; ; Phage_min_cap2 ; Phage minor capsid protein 2(99.92/4.40e-23)<br>COG5585; COG5585; NAD+--asparagine ADP-ribosyltransferase [Signal transduction mechanisms].(99.89/1.90e-21)<br>COG2369; COG2369; Uncharacterized conserved protein, contains phage Mu gpF-like domain [Function unknown].(99.83/5.50e-18)	minor capsid protein
5	4535	5245	+	phage scaffold protein [*Staphylococcus* phage	8.0e-12	-	-	PF14265.7; ; DUF4355 ; Domain of unknown function	DUF4355-containing protein,

				SpaA1]<br>gp8 [*Brochothrix* phage BL3]<br>putative scaffold protein [*Oenococcus* phage phiS11]	3.0e-09<br>9.0e-05			(DUF4355)(99.79/3.70e-17)	putative scaffold protein
6	5262	6251	+	capsid protein [Phage Altai3]<br>coat protein [*Anoxybacillus* phage A403]<br>coat protein [*Enterococcus* phage phiFL3A]	2.0e-156<br>4.0e-143<br>3.0e-99	-	-	PF13252.7; ; DUF4043 ; Protein of unknown function (DUF4043)(99.66/5.00e-15)<br>PF11651.9; ; P22_CoatProtein ; P22 coat protein - gene protein 5(99.37/3.80e-12)<br>d2fsya1; d.183.1.1 (A:104-383) Major capsid protein gp5 {Bacteriophage HK97 [TaxId: 37554]}(99.33/4.90e-11)	major capsid protein
7	6326	6571	+	hp	hp	-	-	PF09124.11; ; Endonuc-dimeris ; T4 recombination endonuclease VII, dimerisation(89.41/1.30e+00)	hp
8	6558	6884	+	putative head morphogenesis protein [uncultured *Caudovirales* phage]<br>head-tail connector protein [*Aeribacillus* phage AP45]	6.0e-09<br>6.0e-08	gp15<br>(29-101)	1.04e-04	PF05135.14; ; Phage_connect_1 ; Phage gp6-like head-tail connector protein(99.7/4.20e-16)<br>cd08051; gp6_gp15_like; Head-Tail Connector Proteins gp6 and gp15, and similar proteins. Members of this family include the proteins gp6	gp15-like head completion protein

								and gp15 from bacteriophage HK97 and SPP1, respectively.(99.69/8.00e-16)<br><br>PF11436.9; ; DUF3199 ; Protein of unknown function (DUF3199)(99.69/4.00e-15)	
9	6884	7510	+	putative morphogenesis protein [*Lactobacillus* phage LBR48]<br>gp11 [*Listeria* phage B054]<br>neck protein [*Vibrio* phage 1.054.O._10N.261.52.A1]	1.0e-34<br><br>9.0e-18<br><br>2.0e-11	-	-	PF05069.14; ; Phage_tail_S ; Phage virion morphogenesis family(98.13/9.20e-06)<br><br>COG5005; COG5005; Mu-like prophage protein gpG [Mobilome: prophages, transposons].(97.26/2.60e-03)<br><br>PF05069.14; ; Phage_tail_S ; Phage virion morphogenesis family(96.57/2.60e-03)	tail completion protein
10	7510	7869	+	hp	hp	-	-	PF12206.9; ; DUF3599 ; Domain of unknown function (DUF3599)(98.95/3.20e-08)<br><br>COG5614; COG5614; Bacteriophage head-tail adaptor [Mobilome: prophages, transposons].(98.94/6.90e-08)<br><br>PF05521.12; ; Phage_H_T_join ; Phage head-tail joining	XkdH-like head completion protein

								protein(98.66/1.30e-06)	
11	7881	8345	+	gp13 [*Listeria* phage B054]	6.0e-04	-	-	PF16807.6; ; DUF5072 ; Domain of unknown function (DUF5072)(96.59/8.10e-02)	hp
12	8361	9368	+	structural protein [*Listeria* phage PSU-VKH-LP041]<br>gp14 [*Listeria* phage B054]<br>tail sheath protein [*Acinetobacter* phage AM24]	1.0e-44<br>1.0e-44<br>1.0e-31	DUF3383<br>(101-333)	2.20e-19	PF11863.9; ; DUF3383 ; Protein of unknown function (DUF3383)(100.0/5.00e-46)<br>COG4386; COG4386; Mu-like prophage tail sheath protein gpL [Mobilome: prophages, transposons].(99.88/1.10e-20)<br>PF10758.10; ; DUF2586 ; Protein of unknown function (DUF2586)(99.67/3.80e-14)	tail sheath protein
13	9381	9788	+	phage structure protein [Deep-sea *thermophilic* phage D6E]<br>structure protein [*Aeribacillus* phage AP45]<br>putative structural protein [*Pseudomonas* phage POR1]	1.0e-45<br>4.0e-17<br>8.0e-09	DUF3277<br>(17-74)	6.23e-07	PF11681.9; ; DUF3277 ; Protein of unknown function (DUF3277)(99.97/1.80e-29)<br>PF07316.12; ; DUF1463 ; Protein of unknown function (DUF1463)(98.39/1.00e-05)<br>d2guja1; b.106.1.3 (A:5-143) Phage-like element PBSX protein XkdM {*Bacillus subtilis* [TaxId: 1423]}(97.15/3.50e-02)	tail tube protein

14	9841	10116	+	-	-	-	-	PF12363.9; ; Phage_TAC_12 ; Phage tail assembly chaperone protein, TAC(89.36/4.00e+00)	putative tail assembly chaperone
15	10148	10333	+	-	-	-	-	PF06528.13; ; Phage_P2_GpE ; Phage P2 GpE(85.69/9.00e-01)	putative tail assembly chaperone
16	10338	13424	+	tape measure protein [*Bacillus* phage 056SW001B]<br>tape measure protein [*Bacillus* phage 019DV002]<br>putative tape measure protein [*Staphylococcus* phage SP5]	4.0e-47<br>4.0e-47<br>8.0e-44	COG5412 (618-905)<br>COG5412 (359-739)	1.70e-12<br>9.46e-07	COG5412; COG5412; Phage-related protein [Mobilome: prophages, transposons].(99.75/1.90e-11)<br>COG5283; COG5283; Phage-related tail protein [Mobilome: prophages, transposons].(99.76/4.00e-09)<br>COG5412; COG5412; Phage-related protein [Mobilome: prophages, transposons].(99.54/6.50e-08)	putative tail tape measure protein
17	13438	14046	+	LysM domain/BON superfamily protein [*Exiguobacterium* phage vB_EauM-23]<br>muramidase [*Aeribacillus* phage AP45]<br>XkdP protein [Clostridium virus phiCD119]	3.0e-25<br>8.0e-14<br>2.0e-08	PRK11198 (145-202)	2.50e-19	COG3499; COG3499; Phage protein U [Mobilome: prophages, transposons].(99.23/1.10e-10)<br>PF06995.12; ; Phage_P2_GpU ; Phage P2 GpU(98.71/2.40e-07)<br>COG1652; XkdP; Nudeoid-associated protein YgaU, contains BON and LysM domains [Function unknown].(98.57/3.60e-07)	LysM domain-containing peptidoglycan-binding protein

18	14059	14376	+	hp	hp	-	-	PF10657.10; ; RC-P840_PscD ; Photosystem P840 reaction centre protein PscD(72.32/6.10e+00)	hp
19	14369	15349	+	gp21 [*Listeria* phage B054]<br>putative baseplate hub [*Enterobacteria* phage ECGD1]<br>Phi92_gp137 [*Enterobacteria* phage phi92]	1.0e-35<br>3.0e-10<br>3.0e-10	-	-	COG4379; COG4379; Mu-like prophage tail protein gpP [Mobilome: prophages, transposons].(99.9/3.70e-21)<br>COG3500; gpD; Phage protein D [Mobilome: prophages, transposons].(99.89/1.50e-20)<br>COG3501; VgrG; Uncharacterized conserved protein, implicated in type VI secretion and phage assembly [Intracellular trafficking, secretion, and vesicular transport, Mobilome: prophages, transposons, General function prediction only].(99.85/3.50e-19)	gpP-like baseplate hub protein
20	15362	15841	+	baseplate central spike protein [*Acinetobacter* phage BS46]<br>putative baseplate protein [*Acinetobacter* phage YMC13/03/R2096]<br>putative spike protein [Prokaryotic dsDNA virus	3.0e-07<br>7.0e-07<br>3.0e-06	Gp138_N<br>(25-108)	1.08e-16	PF18352.2; ; Gp138_N ; Phage protein Gp138 N-terminal domain(99.82/2.60e-19)<br>COG4540; gpV; Phage P2 baseplate assembly protein gpV [Mobilome: prophages, transposons].(99.63/5.60e-14)<br>COG4384; gp45; Mu-like prophage	gpV-like tail spike (tail needle)

				sp.]				protein gp45 [Mobilome: prophages, transposons].(99.56/4.50e-13)	
21	15841	16209	+	putative oxidoreductase [*Burkholderia* phage Bups phi1]<br>putative baseplate component [*Pantoea* phage vB_PagM_AAM37]<br>DUF2634 domain-containing protein [*Listeria* phage PSU-VKH-LP041]	6.0e-06<br>3.0e-04<br>6.0e-04	DUF2634<br>(2-104)<br>GPW_gp25 superfamily<br>(2-104)	9.25e-05<br>2.75e-5	PF10934.9; DUF2634 ; Protein of unknown function (DUF2634) (99.82/8.5e-19)<br>COG4381 gp46; Mu-like prophage protein gp46 [Mobilome: prophages, transposons]. (99.71/9.8e-16)<br>COG3628 COG3628; Phage baseplate assembly protein W [Mobilome: prophages, transposons]. (99.61/8.7e-14)	gp25-like baseplate protein
22	16196	17377	+	baseplate protein [*Aeribacillus* phage AP45]<br>baseplate protein [Deep-sea *thermophilic* phage D6E]<br>putative baseplate protein [uncultured *Caudovirales* phage]	1.0e-71<br>2.0e-69<br>5.0e-61	Baseplate_J<br>(74-188)<br>JayE<br>(6-391)<br>TIGR02243<br>(144-355)	5.98e-23<br>6.40e-22<br>1.99e-05	COG3299; JayE; Uncharacterized phage protein gp47/JayE [Mobilome: prophages, transposons].(100.0/9.00e-37)<br>COG3948; COG3948; Phage-related baseplate assembly protein [Mobilome: prophages, transposons].(99.97/5.50e-32)<br>PF04865.15; ; Baseplate_J ; Baseplate J-like protein(99.97/2.20e-29)	J-like baseplate protein

23	17374	18000	+	gp25 [*Listeria* phage B054]<br>putative baseplate protein [*Lactobacillus* phage LBR48]<br>baseplate protein [*Lactobacillus* phage 3-SAC12]	2.0e-32<br><br>3.0e-15<br><br>1.0e-05	-	-	PF11041.9; ; DUF2612 ; Protein of unknown function (DUF2612)(99.77/2.70e-18)<br><br>PF10076.10; ; DUF2313 ; Uncharacterised protein conserved in bacteria (DUF2313)(99.69/3.20e-15)<br><br>COG3778; YmfQ; Uncharacterized protein YmfQ in lambdoid prophage, DUF2313 family [Mobilome: prophages, transposons].(99.57/4.00e-13)<br><br>PF04865.15; Baseplate_J ; Baseplate J-like protein (97.54/0.00044)	I-like baseplate protein
24	18016	21414	+	tail fiber protein [Deep-sea *thermophilic* phage D6E]<br>tail fiber protein [*Aeribacillus* phage AP45]<br>YomR-like protein [*Bacillus* phage Shbh1]	3.0e-19<br><br>5.0e-17<br><br>6.0e-12	Phage_fiber_2<br><br>(328-365)	4.07e-08	cd16446; TNFSF_C1q; complement component 1, q subcomponent-like (C1ql), a component of C1q/TNF superfamily.(98.32/1.00e-05)<br><br>cd16447; TNFSF_C1q-like; C1q-like family containing adiponectin, and C-terminal NCI domains of collagen VIII and X.(97.85/3.10e-04)<br><br>PF00386.22; ; C1q ; C1q domain(96.97/1.60e-02)	gpH-like tail fiber receptor binding protein

25	21430	21711	+	hp	hp	XkdW(3-88)	6.22e-07	PF09636.11; ; XkdW ; XkdW protein(99.91/5.00e-23)<br>d2hg7a1; d.186.2.1 (A:1-60) Phage-like element PbsX protein XkdW {*Bacillus subtilis* [TaxId: 1423]}(99.7/1.00e-16)	XkdW-like protein
26	21727	21957	+	-	-	-	-	PF09693.11; ; Phage_XkdX ; Phage uncharacterised protein (Phage_XkdX)(97.23/1.20e-03)<br>gp23.1 (SPP1) putative chaperone (97.6/e-6)	putative chaperone
27	22042	22374	+	hemolysin XhlA protein [*Bacillus* phage vB_BtS_BMBtp14]	3.0e-03	XhlA<br>(32-101)	7.74e-10	PF10779.10; XhlA ; Haemolysin XhlA(99.27/1.60e-10)<br>PF07439.12; ; DUF1515 ; Protein of unknown function (DUF1515)(97.59/6.90e-03)<br>PF11166.9; ; DUF2951 ; Protein of unknown function (DUF2951)(97.65/7.60e-03)	XhlA domain-containing holin-like protein
28	22377	23180	+	endolysin [*Sporosarcina* phage Lietuvens]<br>gp22 [*Bacillus* virus G]<br>gp23 [*Bacillus* virus G]	2.0e-72<br>9.0e-70	MurNAc-LAA<br>(7-180)<br>Ami_3	7.22e-40<br>9.65e-21	d1jwqa_; c.56.5.6 (A:) N-acetylmuramoyl-L-alanine amidase CwlV {*Paenibacillus* polymyxa [TaxId: 1406]}(99.97/9.30e-29)<br>COG0860; AmiC; N-acetylmuramoyl-	N-acetylmuramoyl-L-alanine amidase

					2.0e-66	(66-179)		L-alanine amidase [Cell wall/membrane/envelope biogenesis].(99.96/3.40e-27)<br>d1xova2; c.56.5.6 (A:1-180) Endolysin Ply, catalytic domain {Bacteriophage Psa [TaxId: 171618]}(99.95/1.30e-25)	
29	23192	23413	+	SPP1 family holin [*Bacillus* phage Waukesha92]<br>lysis protein [*Bacillus* phage vB_BthS-TP21T]<br>putative holin [*Bacillus* phage PBC4]	4.0e-25<br>2.0e-15<br>6.0e-15	holin_SPP1<br>(1-72)	6.13e-12	PF04688.14; ; Holin_SPP1 ; SPP1 phage holin(99.91/1.00e-23)<br>COG5546; COG5546; Uncharacterized membrane protein [Function unknown].(96.8/3.20e-02)	holin
30	23562	24275	+	hp	hp	-	-	('-', '-')	hp
31	24289	24468	+	-	-	-	-	d1j9ia_; a.6.1.5 (A:) Terminase gpNU1 subunit domain {Bacteriophage lambda [TaxId: 10710]}(98.92/2.20e-08)<br>PF11112.9; PyocinActivator ; Pyocin activator protein PrtN(98.87/2.60e-08)<br>PF10743.10; ; Phage_Cox ; Regulatory phage protein cox(98.85/2.70e-08)	DNA-binding protein

32	24458	24652	+	hp	hp	-	-	('-', '-')	hp
33	24649	24756	+	hp	hp	-	-	PF15168.7; ; TRIQK ; Triple QxxK/R motif-containing protein family(77.99/3.20e+00)	hp
34	24797	25201	+	putative membrane lipoprotein [*Paenibacillus* phage Yerffej]	1.0e-03	-	-	PF12978.8; ; DUF3862 ; Domain of Unknown Function with PDB structure (DUF3862)(99.25/1.70e-10)<br>COG2913; BamE; Outer membrane protein assembly factor BamE, lipoprotein component of the BamABCDE complex [Cell wall/membrane/envelope biogenesis].(99.26/2.30e-10)<br>PF07467.12; ; BLIP ; Beta-lactamase inhibitor (BLIP)(98.96/1.10e-08)	hp
35	25342	26052	+	hp	hp	-	-	('-', '-')	hp
36	26055	26201	+	hp	hp	-	-	PF09538.11; ; FYDLN_acid ; Protein of unknown function (FYDLN_acid)(96.99/3.70e-04)<br>COG1675; TFA1; Transcription initiation factor IIE, alpha subunit [Transcription].(96.83/9.90e-04)<br>PF11023.9; ; DUF2614 ; Zinc-ribbon	hp

								containing domain(95.17/8.10e-03)	
37	26361	26561	+	-	-	-	-	COG1254; AcyP; Acylphosphatase [Energy production and conversion].(95.5/6.70e-02)<br><br>d1apsa_; d.58.10.1 (A:) Acylphosphatase {Horse (*Equus caballus*) [TaxId: 9796]}(95.59/6.80e-02)<br><br>PF00708.19; ; Acylphosphatase ; Acylphosphatase(95.3/7.50e-02)	acylphosphatase
38	26566	26769	+	hp	hp	-	-	PF14122.7; ; YokU ; YokU-like protein, putative antitoxin(73.17/1.90e+00)	hp
39	26913	27200	+	glutaredoxin-like protein [*Bacillus* phage Wes44]<br>glutaredoxin-like protein [*Bacillus* phage Carmen17]	1.0e-43<br><br>4.0e-42	-	-	d1x93a1; a.43.1.3 (A:31-73) Uncharacterized protein HP0222 {*Helicobacter pylori* [TaxId: 210]}(94.42/1.60e-01)<br><br>d1irqa_ a.43.1.4 (A:) Omega transcriptional repressor {*Streptococcus pyogenes* [TaxId: 1314]} (87.73/2.5)<br><br>d2cpga_ a.43.1.3 (A:) Transcriptional repressor CopG {*Streptococcus agalactiae* [TaxId:	CopG/Arc/MetJ DNA-binding domain-containing protein

								1311]} (87.37/2.5)	
40	27193	27549	+	putative glycosyltransferase [*Bacillus* phage vB_BspS_SplendidRed]	3.0e-34	-	-	PF04956.14; ; TrbC ; TrbC/VIRB2 pilin(97.35/3.20e-03)<br>PF18895.1; ; T4SS_pilin ; Type IV secretion system pilin(96.38/6.50e-02)	hp
41	27563	27916	+	hp	hp	-	-	('-', '-')	hp
42	27913	30126	+	ATPase domain protein [*Bacillus* phage 049ML001]<br>ATPase domain protein [*Bacillus* phage 049ML003]<br>ATPase domain protein [*Bacillus* phage 000TH010]	0.0e+00<br>0.0e+00<br>0.0e+00	-	-	d1e9ra_; c.37.1.11 (A:) Bacterial conjugative coupling protein TrwB {*Escherichia coli* [TaxId: 562]}(99.94/1.50e-24)<br>PF19044.1; ; P-loop_TraG ; TraG P-loop domain(99.94/1.70e-24)<br>PF05872.13; ; DUF853 ; Bacterial protein of unknown function (DUF853)(99.92/1.50e-23)<br>PF12846.8; AAA_10 ; AAA-like domain (99.92/6e-23)<br>COG1674 FtsK; DNA segregation ATPase FtsK/SpoIIIE and related proteins [Cell cycle control, cell division, chromosome partitioning].(98.26/0.000018)	ATPase domain protein

43	30454	30594	+	hp	hp	-	-	('-', '-')	hp
44	30769	30909	+	-	-	-	-	d1xrxa1; a.43.1.7 (A:1-35) SeqA {*Escherichia coli* [TaxId: 562]}(94.91/5.10e-02)<br>PF17206.4; ; SeqA_N ; SeqA protein N-terminal domain(94.45/9.30e-02)	putative transcriptional repressor
45	30928	32289	+	exonuclease [*Bacillus* phage Ray17]<br>5'-3' exonuclease [*Bacillus* phage 000TH010]<br>5'-3' exonuclease [*Bacillus* phage 049ML003]	2.0e-87<br>6.0e-87<br>2.0e-85	COG5377<br>(11-322)<br>YqaJ<br>(24-171)<br>PRK00409<br>(313-415)	6.79e-62<br>3.31e-35<br>2.64e-04	COG5377; COG5377; Phage-related protein, predicted endonuclease [Mobilome: prophages, transposons].(100.0/3.50e-40)<br>d3sm4a_; c.52.1.13 (A:) lambda exonuclease {Bacteriophage lambda [TaxId: 10710]}(99.95/7.30e-27)<br>PF09588.11; ; YqaJ ; YqaJ-like viral recombinase domain(99.74/1.90e-17)	YqaJ-domain containing exonuclease
46	32291	32527	+	hp	hp	-	-	PF18077.2; ; DUF5595 ; Domain of unknown function (DUF5595)(95.37/2.10e-01)	hp
47	32529	33374	+	RecT [*Geobacillus* phage GBK2]<br>recombinase [*Bacillus* phage 049ML001]<br>putative recombinase	7.0e-112<br>6.0e-104	recT<br>(23-279)	4.79e-114	COG3723; RecT; Recombinational DNA repair protein RecT [Replication, recombination and repair].(100.0/3.90e-43)	recombination protein RecT

				[*Bacillus* phage vB_BspS_SplendidRed]	2.0e-102	Rect (2-250) RecT (56-253)	2.40e-83 6.86e-51	PF03837.15; ; RecT ; RecT family(100.0/3.70e-31)	
48	33387	33845	+	putative single-stranded DNA-binding protein [*Listeria* phage PSU-VKH-LP019] putative SSB protein [*Listeria* phage A118] gp45 [*Brochothrix* phage NF5]	2.0e-68 7.0e-66 1.0e-64	PRK06751 (1-152) SSB (2-102) Ssb(1-152)	1.58e-70 3.33e-49 2.35e-47	COG0629; Ssb; Single-stranded DNA-binding protein [Replication, recombination and repair].(99.92/1.70e-22) d1qvca_; b.40.4.3 (A:) ssDNA-binding protein {*Escherichia coli* [TaxId: 562]}(99.91/1.20e-21) KOG1653; Single-stranded DNA-binding protein [Replication, recombination and repair](99.84/6.30e-19)	SSBP (single-stranded DNA binding protein)
49	34005	34169	+	hp	hp	-	-	PF10058.10; ; zinc_ribbon_10 ; Predicted integral membrane zinc-ribbon metal-binding protein(70.42/4.50e+00)	hp
50	34220	34873	+	DNA polymerase III subunit beta [*Bacillus* phage Silence] DNA polymerase III	4.0e-60 3.0e-08	beta_clamp (26-215)	3.11e-10	cd00140; beta_clamp; Beta clamp domain. The beta subunit (processivity factor) of DNA polymerase III holoenzyme, refered	PolIIIβ-like processivity factor (DNA clamp)

				subunit beta [*Psychrobacillus* phage Perkons] DNA polymerase III subunit beta [*Staphylococcus* phage SPbeta-like]	4.0e-07	DNA_pol3_beta_3 (126-214) DnaN (79-216)	4.19e-05 8.93e-05	to as the beta clamp, forms a ring shaped dimer that encircles dsDNA (sliding clamp) in bacteria.(99.94/1.70e-24) COG0592; DnaN; DNA polymerase III sliding clamp (beta) subunit, PCNA homolog [Replication, recombination and repair].(99.85/2.30e-19) d1vpka3; d.131.1.1 (A:244-366) DNA polymerase III, beta subunit {*Thermotoga maritima* [TaxId: 2336]}(99.73/2.40e-16)	
51	34901	35428	+	hp	hp	-	-	('-', '-')	hp
52	35428	35754	+	hp	hp	-	-	('-', '-')	hp
53	35756	36040	+	phage related protein [*Staphylococcus* phage SpaA1]	7.0e-14	-	-	PF04009.13; ; DUF356 ; Protein of unknown function (DUF356)(81.87/6.10e+00)	hp
54	36052	36372	+	hp	hp	-	-	COG3462; COG3462; Uncharacterized membrane protein [Function unknown].(78.61/2.70e+00)	hp
55	36373	36546	+	hp	hp	-	-	PF12208.9; ; DUF3601 ; Domain of unknown function	hp

								(DUF3601)(87.76/1.10e+00)	
56	36679	36852	+	DNA-binding protein [*Bacillus* phage Silence]<br>DNA binding domain protein [*Bacillus* phage 049ML001]<br>DNA-binding protein [*Lysinibacillus* phage vB_LspM-01]	9.0e-17<br>3.0e-11<br>1.0e-10	HTH_17<br>(4-52)	1.81e-09	d1j9ia_; a.6.1.5 (A:) Terminase gpNU1 subunit domain {Bacteriophage lambda [TaxId: 10710]}(99.05/1.50e-08)<br>PF11112.9; ; PyocinActivator ; Pyocin activator protein PrtN(99.02/2.10e-08)<br>PF10743.10; ; Phage_Cox ; Regulatory phage protein cox(99.0/2.50e-08)<br>PF06806.13; DUF1233 ; Putative excisionase (DUF1233) (98.72/4.7e-7)<br>d1rh6a_ a.6.1.7 (A:) Excisionase Xis {Bacteriophage lambda [TaxId: 10710]} (98.7/3.1e-7)	DNA-binding protein
57	36852	37055	+	hp	hp	-	-	('-', '-')	hp
58	37064	37858	+	DNA replication protein [*Anoxybacillus* phage A403]<br>replication initiation protein [*Staphylococcus* phage phi879]<br>replication initiation	6.0e-47<br>1.0e-40<br>1.0e-39	-	-	PF09681.11; ; Phage_rep_org_N ; N-terminal phage replisome organiser (Phage_rep_org_N)(98.1/1.20e-04)<br>COG2188; MngR; DNA-binding transcriptional regulator, GntR family [Transcription].(97.37/7.60e-	DnaD domain-containing protein, putative replication initiator

				protein [*Staphylococcus* phage phi575]				04)<br>PF06970.12; ; RepA_N ; Replication initiator protein A (RepA) N-terminus(97.42/8.00e-04)	
59	37869	38261	+	helicase [*Bacillus* phage Ray17]<br>helicase loader [*Bacillus* phage 000TH010]<br>helicase loader [*Bacillus* phage 049ML001]	1.0e-10<br>2.0e-10<br>2.0e-10	Inhibitor_G39P<br>(2-63)	1.82e-04	d1no1a1; a.179.1.1 (A:2-67) Replisome organizer (g39p helicase loader/inhibitor protein) {Bacteriophage Spp1 [TaxId: 10724]}(99.58/1.10e-14)<br>PF11417.9; ; Inhibitor_G39P ; Loader and inhibitor of phage G40P(99.43/6.60e-13)<br>PF06992.12; ; Phage_lambda_P ; Replication protein P(98.91/9.70e-09)	helicase loader
60	38258	39586	+	DNA helicase [*Bacillus* phage Silence]<br>helicase [*Geobacillus* phage GBK2]<br>replicative DNA helicase [*Bacillus* phage 11143]	3.0e-131<br>2.0e-98<br>4.0e-98	DnaB<br>(7-435)<br>DnaB_C<br>(175-433)<br>DnaB	2.73e-139<br>4.61e-115<br>1.33e-111	COG0305; DnaB; Replicative DNA helicase [Replication, recombination and repair].(100.0/4.60e-39)<br>d1cr1a_; c.37.1.11 (A:) Gene 4 protein (g4p, DNA primase), helicase domain {Bacteriophage T7 [TaxId: 10760]}(99.95/7.30e-25)<br>PF03796.16; ; DnaB_C ; DnaB-like helicase C terminal	DnaB-type replicative helicase

						(7-435)		domain(99.95/1.20e-24)	
61	39579	39917	+	HNH endonuclease I [*Bacillus* phage Slash]<br>DNA binding protein [*Bacillus* phage vB_BthS_BMBphi]<br>HNH homing endonuclease [*Bacillus* phage AvesoBmore]	2.0e-23<br>1.0e-19<br>5.0e-19	HNH_3<br>(44-88)	1.66e-13	PF05551.12; ; zf-His_Me_endon ; Zinc-binding loop region of homing endonuclease(99.72/5.90e-17)<br>d1u3em1; d.4.1.3 (M:1-105) Intron-encoded homing endonuclease I-HmuI {Bacteriophage SPO1 [TaxId: 10685]}(99.6/5.00e-14)<br>PF13392.7; ; HNH_3 ; HNH endonuclease(99.38/7.50e-13)	HNH endonuclease
62	40035	40325	+	hp	hp	-	-	PF08858.11; ; IDEAL ; IDEAL domain(98.66/5.40e-08)<br>COG5582; YpiB; Uncharacterized protein YpiB, UPF0302 family [Function unknown].(97.34/5.40e-04)<br>PF09629.11; ; YorP ; YorP protein(95.77/9.90e-02)	hp
63	40401	41510	+	hp	hp	rfaE_dom_I<br>(35-109)	5.93e-04	KOG1319; bHLHZip transcription factor BIGMAX [Transcription](94.47/1.90e+00)	hp
64	41563	41859	+	-	-	-	-	COG3877; COG3877; Uncharacterized protein, DUF2089 family [Function	putative antitermination protein

								unknown].(98.92/4.80e-08)<br>PF07750.12; ; GcrA ; GcrA cell cycle regulator(98.72/5.60e-08)<br>d1ijwc_; a.4.1.2 (C:) HIN recombinase (DNA-binding domain) {Synthetic}(98.03/4.00e-05)	
65	41865	42104	+	transcription factor [*Bacillus* phage Ray17]	3.0e-03	-	-	PF12677.8; ; DUF3797 ; Domain of unknown function (DUF3797)(95.07/3.00e-02)<br>d1twfi1; g.41.3.1 (I:1-49) RBP9 subunit of RNA polymerase II {Baker's yeast (Saccharomyces cerevisiae) [TaxId: 4932]}(95.34/3.40e-02)<br>cd00629; RNA_pol_M_RPB9_N; RNA_pol_M_RPB9_N. RPB9 is a subunit of eukaryotic RNA polymerase II that contributes to transcription elongation by recruiting transcription factor TFIIE to the RNA polymerase II complex.(94.92/6.20e-02)	hp
66	42088	42207	+	hp	hp	-	-	('-', '-')	hp
67	42204	42470	+	glutaredoxin [*Bacillus* phage Claudi]	6.0e-12	NrdH	1.13e-09	d1zmaa1; c.47.1.1 (A:1-115) Bacterocin transport accessory	glutaredoxin-like

				glutaredoxin [*Bacillus* phage DK2]<br>glutaredoxin [*Bacillus* phage DK3]	8.0e-11<br>3.0e-10	(5-86)<br>PRK10329<br>(5-54)	1.41e-04	protein Bta {Pneumococcus (Streptococcus pneumoniae) [TaxId: 1313]}(99.72/5.20e-16)<br>d3diea1; c.47.1.1 (A:1-104) Thioredoxin {*Staphylococcus* aureus [TaxId: 1280]}(99.71/7.80e-16)<br>d1dbya1; c.47.1.1 (A:2-107) Thioredoxin {Green alga (Chlamydomonas reinhardtii) [TaxId: 3055]}(99.71/8.50e-16)	protein
68	42467	42595	+	hp	hp	-	-	COG0690; SecE; Preprotein translocase subunit SecE [Intracellular trafficking, secretion, and vesicular transport].(90.78/2.20e+00)	hp
69	42609	42743	+	hp	hp	-	-	PF16777.6; ; RHH_7 ; Transcriptional regulator, RHH-like, CopG(91.64/6.70e-01)	hp
70	42746	43261	+	putative dUTPase [uncultured *Caudovirales* phage]<br>dUTP diphosphatase [*Aeribacillus* phage AP45]<br>dUTPase [*Bacillus* phage Silence]	2.0e-36<br>1.0e-34<br>4.0e-32	dUTPase_2<br>(2-171)<br>NTP-PPase_dUTPase(6-77)	2.96e-32<br>1.63e-13<br>9.19e-05	COG4508; Dut2; Dimeric dUTPase, all-alpha-NTP-PPase (MazG) superfamily [Nucleotide transport and metabolism].(99.97/2.80e-29)<br>PF08761.12; ; dUTPase_2 ; dUTPase(99.97/3.80e-29)<br>d1w2ya_; a.204.1.1 (A:) Type II	dimeric dUTPase

						56<br>(138-171)		deoxyuridine triphosphatase {*Campylobacter jejuni* [TaxId: 197]}(99.95/5.80e-27)	
71	43320	43490	+	hp	hp	-	-	PF04161.14; ; Arv1 ; Arv1-like family(91.83/5.90e-02)	hp
72	43493	43729	+	hp	hp	-	-	PF13397.7; ; RbpA ; RNA polymerase-binding protein(93.09/7.70e-02)	putative sigma-factor
73	43895	44353	+	gp58 [*Listeria* phage A500]<br>holliday junction resolvase [*Bacillus* phage 276BB001]<br>holliday junction resolvase [*Bacillus* phage 019DV002]	1.0e-26<br>2.0e-23<br>6.0e-23	DUF1064<br>(3-111)	1.62e-35	PF06356.12; ; DUF1064 ; Protein of unknown function (DUF1064)(99.89/2.30e-21)<br>d1m0da_; c.52.1.17 (A:) Endonuclease I (Holliday junction resolvase) {Bacteriophage T7 [TaxId: 10760]}(99.2/4.90e-10)<br>PF05367.12; ; Phage_endo_I ; Phage endonuclease I(99.11/2.80e-09)	DUF1064 domain-containing protein, putative endonuclease
74	44450	44695	+	hp	hp	-	-	cd17794; TetR_C; Tetr-Family Transcriptional Regulator. TetR; This family of bacterial transcriptional repressors is characterized by the short approximately 50 amino acid stretch of residues constituting the helix-turn-helix DNA binding motif,	hp

								around the YRFhY motif.(88.12/4.20e+00)	
75	44692	44865	+	hp	hp	-	-	cd14789; Tiki; Tiki homology domain antagonizes Wnt function via cleavage of amino-terminal residues.(67.25/2.10e+00)	hp

Table S3. Phage genomes used for phylogenetic inference.

№	Name	Genome Accession number	Genome length	GC-content, %	ORFs	Number of tRNAs	BLASTn nucleotide identity to vB_BcM_Sam46, %*	Proteins shared with vB_BcM_Sam46**		ORF number in Sam46 genome and annotation
								number	%	
1	*Bacillus* phage vB_BcM_Sam46	MN604698.1	45,419	41.7	77	0	-	77	-	-
2	*Bacillus* phage vB_BcM_Sam112	MN604230.1	45,037	41.6	75	0	96.0	74	98.7	all except 64 (HNH endonuclease), 65 (hypothetical protein)
3	*Bacillus* phage SPP1	NC_004166.2	44,010	43.7	77	0	3.9	16	20.8	2 (large terminase subunit), 3 (portal protein), 4 (minor capsid protein), 6 (major capsid protein), 16 (putative tail tape measure protein), 28 (N-acetylmuramoyl-L-alanine amidase), 36 (hypothetical protein), 41 (CopG/Arc/MetJ DNA-binding domain-containing protein), 42 (hypothetical protein), 43 (hypothetical protein), 44 (ATPase domain containing protein), 47 (YqaJ-domain containing exonuclease), 49 (recombination protein RecT), 50 (single-stranded DNA binding protein), 62 (helicase loader), 63 (DnaB-type replicative helicase)

4	*Geobacillus* phage GBK2	NC_023612.1	39,078	43.1	61	0	3.2	10	16.4	27 (XhlA domain-containing holin-like protein), 42 (hypothetical protein), 43 (hypothetical protein), 44 (ATPase domain containing protein), 47 (YqaJ-domain containing exonuclease), 49 (recombination protein RecT), 50 (single-stranded DNA binding protein), 62 (helicase loader), 63 (DnaB-type replicative helicase), 72 (dimeric dUTPase)
5	Deep-sea thermophilic phage D6E	NC_019544.1	49,335	46.0	90	0	0.6	20	22.2	3 (portal protein), 4 (minor capsid protein), 8 (gp15-like head completion protein), 9 (tail completion protein), 10 (XkdH-like head completion protein), 11 (hypothetical protein), 12 (tail sheath protein), 13 (tail tube protein), 14 (putative tail assembly chaperone), 17 (LysM domain-containing peptidoglycan-binding protein), 18 (hypothetical protein), 19 (gpP-like baseplate hub protein), 21 (gp25-like baseplate protein), 22 (J-like baseplate protein), 23 (I-like baseplate protein), 28 (N-acetylmuramoyl-L-alanine

										amidase),<br>44 (ATPase domain containing protein),<br>50 (single-stranded DNA binding protein),<br>63 (DnaB-type replicative helicase),<br>72 (dimeric dUTPase)
6	*Geobacillus* virus E2	NC_009552.3	40,863	44.8	66	0	0	4	6.1	28 (N-acetylmuramoyl-L-alanine amidase),<br>50 (single-stranded DNA binding protein),<br>63 (DnaB-type replicative helicase),<br>72 (dimeric dUTPase)
7	*Bacillus* virus 1	NC_009737.2	35,055	44.8	53	0	0	4	7.5	28 (N-acetylmuramoyl-L-alanine amidase),<br>47 (YqaJ-domain containing exonuclease),<br>49 (recombination protein RecT),<br>72 (dimeric dUTPase)
8	*Geobacillus* phage GBSV1	NC_008376.2	34,683	44.4	55	0	0	4	7.3	28 (N-acetylmuramoyl-L-alanine amidase),<br>47 (YqaJ-domain containing exonuclease),<br>49 (recombination protein RecT),<br>72 (dimeric dUTPase)
9	*Thermus* phage phi OH2	NC_021784.1	38,099	44.7	63	0	1.3	6	9.5	1 (FtsK gamma domain-containing small terminase subunit),<br>2 (large terminase subunit),<br>3 (portal protein),<br>47 (YqaJ-domain containing exonuclease),<br>49 (recombination protein RecT),<br>72 (dimeric dUTPase)

10	Bacteriophage Lily	NC_028841.1	44,952	42.7	73	0	0	2	2.7	61 (DnaD domain-containing protein, putative replication initiator), 63 (DnaB-type replicative helicase)
11	*Paenibacillus* phage Vegas	NC_028767.1	45,653	43.6	75	0	0	0	-	-
12	*Paenibacillus* phage Tripp	NC_028930.1	54,439	48.3	90	0	0	1	1.1	28 (N-acetylmuramoyl-L-alanine amidase)
13	*Bacillus* virus Eoghan	NC_020477.1	49,458	42.2	79	0	0	3	4.0	28 (N-acetylmuramoyl-L-alanine amidase), 63 (DnaB-type replicative helicase), 75 (DUF1064 domain-containing protein, putative endonuclease)
14	*Bacillus* virus Taylor	NC_041858.1	49,492	42.3	81	0	0	3	3.7	
15	*Bacillus* virus Blastoid	NC_022773.1	50,354	42.2	83	0	0	3	3.6	
16	*Bacillus* virus Finn	NC_020480.1	50,161	41.7	83	0	0	3	3.6	
17	*Bacillus* virus Riggi	NC_022765.1	49,836	41.5	82	0	0	3	3.5	
18	*Bacillus* phage Gemini	KC330681.1	49,362	41.9	80	0	0	3	3.8	
19	*Bacillus* virus Andromeda	NC_020478.1	49,259	41.9	80	0	0	3	3.8	
20	*Bacillus* virus Glittering	NC_022766.1	49,246	42.1	83	0	0	3	3.6	
21	*Bacillus* virus Curly	NC_020479.1	49,425	41.8	81	0	0	3	3.7	
22	*Bacillus* phage PM1	NC_020883.1	50,861	41.3	92	0	0	4	4.3	2 (large terminase subunit), 28 (N-acetylmuramoyl-L-alanine amidase), 50 (single-stranded DNA binding protein), 59 (DNA-binding protein)

*Determined using BLASTn compared to vB_BcM_Sam46 (multiplying % coverage by % identity); **Determined using GET_HOMOLOGUES (COGtriangles algorithm, -t 0 -C 75).

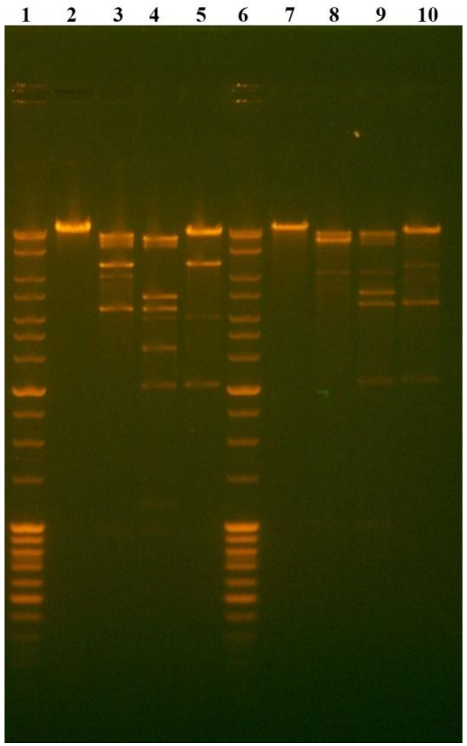

Fig. S5. Restriction analysis of Sam46 and Sam112 DNA with enzymes XbaI and HindIII. This is the original gel image used to generate Figure 7b in the main text. Kodak EDAS 290 Gel Documentation System ("Kodak") was used to capture the image. 1 and 6 – molecular weight markers; 2 and 7 – intact phage DNA, 3 and 8 – HindIII, 4 and 9 – HindIII + XbaI, 5 and 10 – XbaI.
